# Predicting the DNA binding specificity of transcription factor mutants using family-level biophysically interpretable machine learning

**DOI:** 10.1093/nar/gkaf831

**Published:** 2025-08-28

**Authors:** Shaoxun Liu, Pilar Gomez-Alcala, Christ Leemans, William J Glassford, Lucas A N Melo, Xiang-Jun Lu, Richard S Mann, Harmen J Bussemaker

**Affiliations:** Department of Biological Sciences, Columbia University, New York, NY 10027, United States; Department of Biological Sciences, Columbia University, New York, NY 10027, United States; Department of Biological Sciences, Columbia University, New York, NY 10027, United States; Department of Biochemistry and Molecular Biophysics, Columbia University, New York, NY 10032, United States; Department of Biological Sciences, Columbia University, New York, NY 10027, United States; Department of Biological Sciences, Columbia University, New York, NY 10027, United States; Department of Biochemistry and Molecular Biophysics, Columbia University, New York, NY 10032, United States; Department of Systems Biology, Columbia University, New York, NY 10032, United States; Department of Biological Sciences, Columbia University, New York, NY 10027, United States; Department of Systems Biology, Columbia University, New York, NY 10032, United States

## Abstract

Sequence-specific interactions of transcription factors (TFs) with genomic DNA underlie many cellular processes. High-throughput *in vitro* binding assays coupled with machine learning have made it possible to accurately define such molecular recognition in a biophysically interpretable way for hundreds of TFs across many structural families, providing new avenues for predicting how the sequence preference of a TF is impacted by disease-associated mutations in its DNA binding domain. We developed a method based on a reference-free tetrahedral representation of variation in base preference within a given structural family that can be used to accurately predict the effect of mutations in the protein sequence of the TF. Using the basic helix-loop-helix (bHLH) and homeodomain (HD) families as test cases, our results demonstrate the feasibility of accurately predicting the shifts (ΔΔΔ*G*/*RT*) in binding free energy associated with TF mutants by leveraging high-quality DNA binding models for sets of homologous wild-type TFs.

## Graphical Abstract

**Graphical Abstract:**
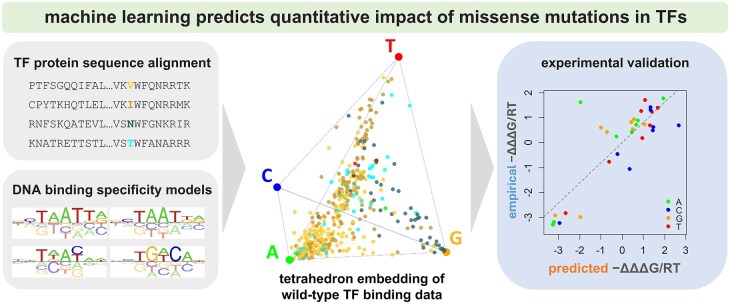

## Introduction

Gene expression regulation, mediated to a significant extent by transcription factors (TFs), is a central aspect of cellular function that is especially important during cellular differentiation and stress responses. The DNA binding domain of a TF enables it to perform its regulatory function by recognizing and binding to specific DNA sequences [1]. TF binding sites typically reside either in proximal promoter regions near transcription start sites or in more remote enhancer regions. By binding to these sites, TFs regulate gene expression, frequently by recruiting transcriptional co-activators or co-repressors [2]. The range of TF binding affinity—typically represented by a dissociation constant (K_D_), which equals the free TF concentration at which the DNA is bound 50% of the time—covers many orders of magnitude between optimal and non-specific binding, and even ultra-weak binding sites can be functionally relevant [3–6]. It is therefore of great value to be able to predict DNA binding affinity over its full range, and understand how this affinity changes when TF binding sites are mutated [7].

Previous studies have provided evidence that mutations in TFs are responsible for numerous common developmental disorders. For example, anophthalmia has been linked to mutations in SOX2 [8], while autosomal dominant Rolandic epilepsy is associated with mutations in NEUROG1 [9]. A single-residue mutation in the DNA binding domain of TFs has the potential to disrupt their DNA binding preference, leading to dysregulated developmental pathways [10, 11]. In addition to disrupting binding, TF mutations can cause quantitative shifts in DNA binding affinity, which can be functionally relevant even when the preferred base remains the same [12]. Detecting such subtle changes requires quantitative protein-DNA binding assays such as protein binding microarrays (PBM) [13], SELEX-seq/HT-SELEX [14–16] or chromatin immunoprecipitation coupled with deep sequencing (ChIP-seq) [17]. These assays, however, are both labor-intensive and expensive: PBM and SELEX-seq involve *in vitro* protein purification; ChIP-seq requires manual collection of tissues and immunoprecipitation with antibodies that can vary widely in their efficacy. As a consequence, with a few exceptions (e.g. [18]), their application has mostly been limited to wild-type TFs.

When modeling the DNA binding specificity of an individual TF, an approximation that treats the effects of base-pair mutations at different positions within the DNA binding site as independent is often relied upon [19–22]. This is equivalent to assuming additivity of binding free energies. For a given TF, the binding specificity model takes the form of a position-specific matrix containing the free energy differences (ΔΔ*G* in units of RT) associated with base substitutions at each DNA position. In previous work, we developed computational methods for estimating ΔΔ*G*/*RT* parameters from high-throughput TF binding data with sufficient accuracy to make predictions of low-affinity binding sites functionally meaningful [6, 22, 23].

Several previous studies have analyzed variation in DNA binding specificity among wild-type TFs within specific structural families [24–28]. Having accurate models would facilitate the characterization of TF variants, as it would greatly reduce the number of variants that need to be tested experimentally. Recent large-scale efforts to empirically determine the impact of disease-associated missense mutations in TF protein sequences on DNA binding specificity [18, 29] have created a new opportunity to benchmark the predictive performance of such family-level predictive modeling.

In this study we present a method for constructing a quantitative *FamilyCode* from DNA binding specificity models for a collection of wild-type TFs from the same protein family that is capable of making accurate predictions of shifts in base preference associated with missense mutations in TFs from the same structural family. Our approach requires as inputs the protein sequences of a set of wild-type TFs from the same family, along with data from suitable DNA binding assays on each of them. We build on the interpretable machine learning framework of FeatureREDUCE [30] for PBM data and ProBound [22] for SELEX-seq data, which allows us to accurately estimate the ΔΔ*G*/*RT* parameters that define a TF’s base preferences using data from *in vitro* binding assays. To analyze the quantitative relationship between base preference and TF protein sequence within a given TF family, we use an innovative reference-free tetrahedron representation of base preference. This enables us to systematically map the protein features that are the most important determinants of differences in DNA binding specificity among TF paralogs. As a proof of concept, we focus on two distinct TF families: basic helix-loop-helix (bHLH) proteins, which are widely studied in both eukaryotes and bacteria [31], and homeodomain (HD) proteins, which play major developmental roles in eukaryotes [32]. To rigorously assess the predictive performance of our FamilyCode models, we performed our own SELEX-seq assays on mutated helix-loop-helix (bHLH) factors, and also used recently published PBM data for mutant bHLH [33] and homeodomain (HD) [29] TFs.

## Materials and methods

### Collecting HT-SELEX data for training bHLH binding models

The training set data used in this study consisted of HT-SELEX data for all TFs annotated as belonging to the bHLH family from three publications of the Taipale lab [16, 34, 35]. For the data from Yin *et al.*, we only used the unmethylated library and ignored reads corresponding to methylated DNA ligands. This yielded a total of 121 multi-round HT-SELEX datasets covering 62 distinct bHLH proteins.

### Constructing binding free energy models using ProBound

For each of the 121 HT-SELEX datasets, we ran ProBound (github.com/RubeGroup/ProBound and Ref. [22]) using the JSON configuration file in Supplemental Data SD1, which let us fit a single binding free energy model to data from all rounds of the SELEX assay simultaneously. We then filtered the models by model quality based on the following two heuristic criteria: (i) sufficient ability of the model to predict sequence enrichment at the level of 8-mers (R^2^ > 0.15), and (ii) a base preference pattern consistent with the E-box consensus CANNTG in the center of the binding free energy model. A total of 97 models passed these criteria. A bHLH factor was assigned the binding model with the highest R^2^ value if multiple models for it passed the quality filter. Our final bHLH binding model compendium comprised 52 distinct bHLH proteins.

### Mapping of DNA binding free energy (ΔΔ*G*/*RT*) parameters to tetrahedral coordinates

In the position-specific affinity matrix (PSAM) representation for a given TF [36], each column $j$ corresponds to a different position within the DNA binding site, each row $b$ to a different base, and each element ${{w}_{jb}} = {\mathrm{exp}} ({-{\mathrm{\Delta \Delta }}{{G}_{jb}}/RT} )$ to the affinity relative to the preferred base ${{b}_0}( j )$ at position $j$ (with the convention that ${{w}_{j{{b}_0}( j )}} = 1$, or equivalently ${\mathrm{\Delta \Delta }}{{G}_{j{{b}_0}( j )}} = 0,$ for the preferred base at each position). To map the four relative affinities in a column of the PSAM to a 3D position within the tetrahedral embedding space, the relative affinities are first normalized to frequencies ${{f}_{jb}} = \ {{w}_{jb}}/( {\mathop \sum \limits_{b^{\prime}} {{w}_{jb^{\prime}}}} )$. In a subsequent transformation step, the transpose of the frequency matrix is multiplied by a 4 × 3 tetrahedral transformation matrix:


\begin{eqnarray*}
{{v}_j} = \ \left( {{{v}_{jx}}\ {{v}_{jy}}\ {{v}_{jz}}} \right) = \ \left( {{{f}_{jA}}\ {{f}_{jC}}\ {{f}_{jG}}\ {{f}_{jT}}} \right) \ \left( {\begin{array}{rrr} 1 & 1 & 1 \\ 1 & -1 & -1 \\ -1 & 1 & -1 \\ - 1 & -1 & 1 \end{array}} \right)
\end{eqnarray*}

This operation maps each PSAM column containing four elements to the Cartesian coordinates $( {x,y,z} )$ within a tetrahedron. The tetrahedron has corners located at positions $( {1,\ 1,\ 1} )$, $( {1,\ - 1,\ - 1} )$, $( { - 1,\ 1,\ - 1} )$, and $( { - 1,\ - 1,\ 1} )$, corresponding to a strict preference for A, C, G, and T, respectively—i.e. ${{w}_{jb}} = 0$ for all $b \not= {{b}_0}( j )$. Any set of non-negative values ${\mathrm{\Delta \Delta }}{{G}_{jb}}/RT$ maps to a 3D position inside the tetrahedron. This mapping is non-linear, especially when ${\mathrm{\Delta \Delta }}{{G}_{jb}}/RT$ becomes numerically large when ${{w}_{jb}} \ll 1$ for any base $b$ at position $j$, reflecting a strong preference against that base. In this regime, when the mapped position within the tetrahedron is near its vertices, faces, or edges, it becomes less sensitive to the precise value of ${\mathrm{\Delta \Delta }}{{G}_{jb}}/RT$. This naturally alleviates the numerical issues associated with ΔΔ*G*/*RT* for disfavored base/position combinations, whose estimates from the data tend to be less precise due to the lower signal-to-noise ratio associated with weaker DNA binding (Supplementary Fig. S1A and B).

### Inverse mapping of tetrahedral coordinate shifts to ΔΔΔ*G*/*RT* values

The tetrahedral mapping itself is invertible, in the sense that any 3D position within the tetrahedron can be readily transformed back into relative affinities $w$ or relative binding free energy parameters ${\mathrm{\Delta \Delta }}G/RT$ using a reverse matrix transformation. However, in our family-level modeling we also consider 3D *shifts* from one internal tetrahedral position to another. In principle, the vector connecting two points can be placed at the origin $( {0,0,0} )$ of the tetrahedral embedding space, and the inverse mapping could be applied to its end point to obtain ${\mathrm{\Delta \Delta \Delta }}G/RT$, the shift in free energy parameters associated with the missense mutation. However, when the changes in base preference are large, the end point of the shift vector may lie outside the tetrahedron. This needs to be remedied before the inverse mapping can be applied. To this end, we used a procedure that moves the point along the line connecting it with the vertex corresponding to the least-preferred base to where the line intersects with the plane that represents a relative affinity of 0.01 for that base (Supplementary Fig. S1C). If needed, this procedure is repeated until the point is fully inside the tetrahedron.

### Alignment of bHLH protein sequences

The cloned protein sequences of the bHLH transcription factors whose HT-SELEX data we trained on were obtained from Refs. [16, 34, 35]. They were individually aligned to the hidden Markov model (HMM) for PFAM entry PF00010 (www.ebi.ac.uk/interpro/entry/pfam/PF00010/) [37, 38]. This allowed their amino acid identities to be mapped to standardized residue positions in the range 1–53.

### Multivariate analysis of variance (MANOVA)

Multivariate analysis of variance (MANOVA) is a generalization of the t-test to deal with multiple groups of indepent variables and multiple dependent variables at the same time. A series of MANOVA tests were performed to examine the association across all bHLH factors between amino acid identity (independent variable) at a given residue position in the protein alignment and tetrahedral position (dependent variable) reflecting the base preferences for a given position in the DNA binding model. For each combination of residue position and DNA position, we used the summary.manova() function in R, with the F-statistic computed using the Pillai–Barlett trace test. The null hypothesis is that the mean tetrahedral position is the same for all amino acids.

### bHLH replicate comparisons used as reference for predictions

Of the 52 unique bHLH proteins, 36 had two replicates available (when more than two replicates were available, we chose the two with best correlation between predicted and observed 8-mer enrichment). The ΔΔ*G*/*RT* estimates from the binding model for these replicates were compared to obtain a reference for predictive performance.

### Definition of closest-paralog for predicting binding specificity

For each of the 52 bHLH proteins that was held out, the protein with the smallest Levenshtein distance in terms of protein sequence (equivalent of percentage amino acid identity) was selected from the remaining 51 sequences. The ΔΔ*G*/*RT* estimates from the binding model for this closest-paralog were then used as predicted values.

### Implementation of similarity regression prediction

Pre-trained positional weights are adopted from the original similarity regression paper for the bHLH and HD families [39]. For the bHLH family, two gaps were added between the 34^th^ and 35^th^ position of the bHLH alignment to match the alignment length recorded in the similarity regression study. The difference in alignment length is likely caused by usage of different HMM models to represent the family. The most highly represented motif is selected by multiplying the amino acid identity profile with the pre-trained weights at each position, deriving a weighted similarity score. The motif associated with the protein sequence that has the smallest similarity score is selected as inferred motif.

### Construction of SVD-regression model

To perform SVD regression for a given DNA position, we first constructed a tetrahedral coordinate matrix $M$ whose three columns correspond to the coordinates of the 3D space in which the tetrahedron is embedded, and whose rows correspond to the set of bHLH proteins analyzed. The 52 × 3 matrix $M$ was centered by subtracting the column mean from each value. Next, we performed singular value decomposition (SVD): $M = UD{{V}^T}$using the base function svd() in R. The columns of 3 × 3 matrix $V$ define a natural data-driven basis for the vector space inside the tetrahedron, while the columns of U correspond to the projection of the points inside the tetrahedron along each of these principal component (PC) directions.

For each of the three PCs (*c*= 1,2,3) for a given DNA position, we separately constructed a linear regression model that predicts the position of an unseen bHLH factor from its protein sequence. Each residue position *r* in the bHLH multiple alignment corresponds to a feature that could be used as a predictor. The importance of each of the 159 combinations among 53 residue positions and 3 PCs was assessed using an ANOVA test implemented using the aov() function in R, with the amino acid identity *a* as the independent variable and the values in each column of $U$ as the dependent variable. Each residue position is associated with three p-values, one for each PC. For each PC, we ranked the protein features by their ANOVA p-value, and iteratively selected for the features to include. For each principal component *c* we started by fitting a linear regression model based on the most statistically significant residue position *r*, in which the position of each transcription factor *f* along *c* for the centered matrix *M* was predicted to be proportional to the mean position of all TFs with the same amino acid at position *r*:


\begin{eqnarray*}
{{m}_{fc}} = {{\beta }_{cr}}\mathop \sum \limits_a \delta \left( {a,a\left( {f,r} \right)} \right)\frac{1}{{\left| {{{F}_{ra}}\left( f \right)} \right|}}\mathop \sum \limits_{f^{\prime} \epsilon {{F}_{ra}}\left( f \right)} {{m}_{f^{\prime}c}}
\end{eqnarray*}

Here, ${{\beta }_{cr}}$ denotes a regression coefficient, $\delta ( {a,a{\mathrm{^{\prime}}}} ) = 0,1$ is an indicator function that is equal to 1 when $a = a{\mathrm{^{\prime}}}$ and 0 otherwise, $a( {f,r} )$ is a lookup function that provides the amino acid identity at residue position *r* for factor *f*, and ${{F}_{ra}}( f )$ is the set of all TFs *except f* that have amino acid *a* at position *r*. Next, we compared with a linear model using an additional feature. As long as the *P*-value of the *F*-test was below 0.05, indicating a significant improvement in variance explained, we included the additional feature in our model and proceeded to the next feature. After the iterative feature selection step, residue positions selected for each PC will have coefficients associated with them that get estimated using linear regression, resulting in three models, one for each of the three PCs. When making predictions for an unseen TF, the mean PC value across all TFs in the training set with the same amino acid at a given position is used.

For amino acids unseen in the training set at a given position, the PC value is estimated as a mean across all amino acids in the training set weighed by the inverse of the difference between the identity score and mismatch score in the BLOSUM62 matrix [40]. The encoded values of each residue position are multiplied by the coefficient matrix to generate predicted PC values. These are in turn transformed into tetrahedron coordinates using a reverse SVD transformation, and mapped to a PSAM using the inverse operation of the tetrahedron embedding. For cases where the resulting PSAM has one or more negative values, they are set to 0.01. Finally, the predicted PSAM is transformed to –ΔΔ*G*/*RT* values by taking the natural logarithm of the relative affinities.

### Leave-one-out cross-validation with sequence distance thresholds

For standard leave-one-out cross-validation, one of the TFs was held out as the test set, and the motif models and protein sequences for the remaining TFs were used as the training set to construct the prediction model. This was done similarly for each method (FamilyCode, closest-paralog, and similarity regression). For leave-one-out cross-validation with distance threshold, the hamming distance between the protein sequence of the held-out TF and each of the remaining protein sequences was computed, and TFs in the training set for which this hamming distance was smaller than the threshold were excluded from the training set.

### Predicting ΔΔΔ*G*/*RT* for TFs mutants using tetrahedral regularization

To predict the effect of TF point mutations on DNA binding specificity, two modifications are made to the SVD regression model. Firstly, during feature selection, protein features that represent the mutated positions are designated as key features, and iterative feature selection is not used. Secondly, instead of directly predicting the –ΔΔ*G*/*RT* values for the mutant TF and subtracting the −ΔΔ*G*/*RT* values for the corresponding wild-type TF, we calculated the shift between the mutant and wild-type TF in tetrahedron space and transformed the difference back to energy space to obtain ΔΔΔ*G*/*RT* values, using the same transformation procedures as used for the construction of SVD-regression model above (Supplementary Fig. S2).

### Application to PBM data from the CisBP database

To train the PCA-regression model on PBM data, PWMs, TF metadata, and normalized fluorescent intensities (Z-scores) were downloaded from the CisBP database (cisbp.ccbr.utoronto.ca) for both the bHLH and HD protein families. To analyze the motif models in CisBP, we directly imported all PWMs from the database and scored each motif model with the corresponding motif seed (CANNTG for bHLH, NNTDAYNN for HD) and collected the best-matching k-mer motif models. For PBM entries for which z-scores were available, we constructed a binding model with PyProBound v1.5.0 (github.com/BussemakerLab/PyProBound) [23], using NCANNTGN and NNTDAYNN as an 8-mer seed for bHLH and HD, respectively. We then searched for the sequence of the longest isoform of each unique protein from Uniprot [41], and aligned it to the HMM of the bHLH and HD families from PFAM [38]. A detailed description of the analyses with reproducible R scripts is available at github.com/BussemakerLab/FamilyCode and doi.org/10.5281/zenodo.15858756.

### Application to homeodomain data

Binding affinity models built from HT-SELEX data for HD factors using ProBound were obtained from MotifCentral.org [22]. When using PyProBound to infer binding models from PBM data, as a pragmatic way to impose a consistent “binding frame” on the models, we aligned each of the resulting HD models to the NNTDAYNN 8-mer IUPAC consensus motif for HDs (here D matches A, C, or T; Y matches the pyrimidines C or T only, and N matches any base) by optimizing a motif matching score (MMS) defined as follows:


\begin{eqnarray*}
{\mathrm{MMS}} = \ \sqrt {\left( {\frac{1}{J}} \right)\mathop \sum \limits_{\left( {j,b} \right)\ \epsilon \ {\mathrm{IUPAC}}} \mathop {\min }\limits_{b^{\prime}} {{{\left( {1 - \ {\mathrm{PSA}}{{{\mathrm{M}}}_{j,b^{\prime}}}\ } \right)}}^2}}
\end{eqnarray*}

Here *J* is the length of the motif seed consisting of a 4x*L* matrix representing the IUPAC identification of the motif. The four rows of the matrix represent the four bases, and each column represents a motif position. Each position in the matrix is set to unity if the IUPAC symbol covers the given base, and zero otherwise. The protein sequences of the HD proteins were retrieved as the recorded cloned sequences, and aligned to the HMM model for PF00046 (www.ebi.ac.uk/interpro/entry/pfam/PF00046/) [37, 38] using the same procedure as for the bHLH family. Note that with this definition of residue position, the traditional Arg51 based on the Engrailed sequence [42] is now Arg50 (Fig. 5A).

### Analysis of PBM data for mutant homeodomains using PyProBound

We obtained protein-binding microarray (PBM) data for 92 single-residue mutants of human homeodomain (HD) proteins and their corresponding wild-type HDs from Ref. [29]. Raw intensity data were processed using PyProBound to generate ΔΔ*G*/*RT* models. The quality of each model was assessed by calculating the coefficient of determination (*R*²) between predicted and empirical probe intensities [22, 30, 43]. Of the 92 mutants reported, 89 had at least two replicates. For each mutant, the replicate with the highest *R*² was designated as the replicate 1, and the replicate with the second-highest R² as replicate 2. Empirical ΔΔΔ*G*/*RT* values were determined for each mutant replicate by comparing its ΔΔ*G*/*RT* model to its wild-type counterpart using the tetrahedron regularization. The *R*^2^ and RMSD between the ΔΔΔ*G*/*RT* values for the respective replicates served as a measure of empirical reproducibility.

### Using FamilyCode to make ΔΔΔ*G*/*RT* predictions for homeodomains

We used a methodology similar to how we evaluated the HLH-1 mutants. A FamilyCode model was trained on a compendium of ΔΔ*G*/*RT* binding specificity models for wild-type HDs derived from a mix of SELEX and PBM data, and used to predict ΔΔΔ*G*/*RT* values quantifying base preference shifts for each of the 92 mutant HDs from Ref. [29]. Empirical ΔΔΔ*G*/*RT* values were computed by using the tetrahedron representation to compare the ΔΔ*G*/*RT* model derived from the PBM data [29] for each mutant HD with its wild-type counterpart. Prediction accuracy was evaluated by computing the *R*² and RMSD between predicted and empirical ΔΔΔ*G*/*RT* values across all eight motif positions.

### Comparison with rCLAMPS

The rCLAMPS tool was implemented following instructions from its GitHub repository [44]. Full-length sequences of each HD protein from the study were retrieved from UniProt [41], selecting the longest isoform, and point mutations were introduced as specified. A FASTA file containing the mutant sequences was input into the pretrained rCLAMPS model, yielding 92 PWM motif models. These models were used to calculate ΔΔΔ*G*/*RT* values and evaluated in the same manner as our FamilyCode models with the slight modification of using 6 nucleotide positions instead of 8, as rCLAMPS only predicts binding specificity to the TDAYNN 6-mer motif. To assess the difference between the prediction accuracy of the FamilyCode model and the rCLAMPS model, we performed a paired Wilcoxon signed-rank test on the 92 *R*^2^ values.

### Comparison with DeepPBS

Unlike FamilyCode and rCLAMPS, the DeepPBS method [45] relies on structural information to predict DNA binding specificity for unseen TFs. We obtained full-length sequences of mutant HD proteins from UniProt, selected the longest isoform, and used the same DNA sequence as an initial guess for HD proteins (GCGTGTAAATGAATTACATGT) as in Ref. [45]. These sequences were submitted to the AlphaFold3 server [46] to predict the structures of the protein–DNA complexes. Model_0 from each prediction was then submitted to the DeepPBS server with the “both readout” option to obtain PWM predictions. The predicted motifs were aligned to the NNTDAYNN 8-mer core using the same method we used for the HD training data alignment. The resulting motif models were used to calculate ΔΔΔ*G*/*RT* values using the tetrahedron representation.

### Wild type and mutant HES2 and ASCL2 protein expression

ASCL2 and HES2 DNA binding domain (DBD) sequences were expressed based on the cloned sequences described in Yin *et al.* [35], tagged with an N terminal 6xHis tag, and expressed and purified according to the protocol described in Slattery *et al.* [47]. To create mutant proteins, site-directed PCR was performed using primers containing mutations at the 5th and 13th residue positions. For HES2, the wild type and mutant sequences were cloned into the pQE30 vector (Qiagen). ASCL2 wild type and mutants were tagged with mScarlet to enhance solubility [48], and were cloned into the pET11 vector (Novagen). The full mScarlet sequence is as follows:

VSKGEAVIKEFMRFKVHMEGSMNGHEFEIEGEGEGRPYEGTQTAKLKVTKGGPLPFSWDILSPQFMYGSRAFIKHPADIPDYYKQSFPEGFKWERVMNFEDGGAVTVTQDTSLEDGTLIYKVKLRGTNFPPDGPVMQKKTMGWEASTERLYPEDGVLKGDIKMALRLKDGGRYLADFKTTYKAKKPVQMPGAYNVDRKLDITSHNEDYTVVEQYERSEGRHSTGGMDELY

All protein samples are expressed in BL21 *Escherichia coli* cells. The BL21 cell are grown in LB plus 100 μg/ml carbenicillin for 2.5 h before IPTG induction and continues to express proteins for ∼4.5 h at 37°C. The cells are then collected by centrifugation and resuspended in 8ml of lysis buffer (50 mM Tris pH 7.5, 600mM NaCl, 40 mM Imidazole). The lysate then went through sonication for five rounds of 30 s with 1-min intervals. The lysate was then spun down and proteins in the supernatant are purified with affinity purification using Cobalt–Talon beads (Clontech) using a low imidazole wash buffer (50 mM Tris pH 7.5, 600 mM NaCl, 20 mM Imidazole), and collected with a high imidazole elution buffer (50 mM Tris pH 7.5, 600 mM NaCl, 300 mM Imidazole). Protein samples are dialyzed in dialysis buffer (20 mM HEPES pH 7.9, 200 mM NaCl, 10% Glycerol, 2 mM MgCl_2_) overnight before changing dialysis buffer and another 2-h dialysis. All procedures above are performed at 4°C. The final concentration of HES2 obtained was 17 ± 3 μM for wild type and R5K mutant and 70 ± 20 μM for R13V and double mutant; the final concentrations obtained were 14 ± 1 μM for ASCL2 wild type and mutants.

### SELEX assays

SELEX experiments were performed following the protocol outlined in Riley *et al.* [14] and a SELEX library with a 16-mer randomized region whose full sequence is as follows:

GTTCAGAGTTCTACAGTCCGACCTAA-16N-TTAGGACTCGGACCTGGACTAGG

The libraries were double strand DNA annealed and extended with Klenow polymerase. The SELEX annealing primers are as follows:

SELEX-L: GTTCAGAGTTCTACAGTCCGA

SELEX-R: CCTAGTCCAGGTCCGAGT

To be specific, the initial mixture was prepared in a DNA LoBind Safe lock tube (Eppendorf 022 431 021) by combining 10 μL of 10x STE buffer (100 mM Tris pH8.0, 10 mM EDTA pH8.0, 1M NaCl), 10 μL of 100 μM SELEX library oligo, 20 μL of 100 μM annealing primer (sequence), and 60 μL of water. The tube was then placed in a 1-liter beaker containing 800 mL of water and boiled for 10 min. After boiling, the tube was left in the hot water until the water cooled to room temperature naturally. Next, 100 μL of the annealed DNA from the first step was taken and mixed with 25 μL of 10x Klenow buffer, 20 μL of 10 mM dNTP, 80 μL of water, and 20 μL of Klenow fragment (NEB M0210L). The mixture was incubated at room temperature for 30 min. To stop the reaction, 10 μL of 0.5M EDTA at pH 8.0 was added. Following this, 25 μL of 3M sodium acetate at pH 5.2 was added, and the DNA was purified using a Qiagen column (Qiagen 28 106) according to the regular PCR purification protocol.

With the SELEX libraries ready, an EMSA gel was prepared to separate the bound DNA fragments from the unbound. To prepare a gel for EMSA, 3.5 ml of 5 × TBE, 3.1 ml of 30% acrylamide/bis-acrylamide solution (37.5:1), 2.3 ml of 40% acrylamide solution, and 1.1 ml of 80% glycerol were added to 24.25 ml of water. The mixture was mixed well, avoiding the generation of bubbles. Then, 262.5 ml of 10% ammonium persulfate and 17.5 mL of TEMED were added to catalyze polymerization. The solution was briefly mixed, poured into a mold to form a gel, and allowed to solidify for 1 h. After solidification, the wells of the gel were flushed with 0.5 × TBE to remove unpolymerized acrylamide, and the gel was pre-run for 10 min at 150 V in 0.5 × TBE buffer. The gel was run in a cold room at 4°C.

Fluorescent EMSA assays were performed alongside the SELEX reactions to define the position of the bound band in the gel. The high-affinity probes for HES2 and ASCL2 binding, respectively, were as follows (E-box bolded, sequence difference underlined):

HES2: CTCTCCTCCGTCAA-**CACGTG**-TTGAGCAGCGCAGTCGTATGCCGTCTTCTGCTTG

ASCL2: CTCTCCTCCGTCAA-**CAGCTG**-TTGAGCAGCGCAGTCGTATGCCGTCTTCTGCTTG

For the Fluorescent EMSA reactions, 30mL reactions were set up as follows: 9 μL of labeled control probe at 166.67 nM final concentration diluted with water, 15 μL of ASCL2 wild type or HES2 wild type protein at 300 nM final concentration diluted with dialysis buffer, and 6 mL of 5 × binding buffer (50mM Tris pH 7.5, 250mM NaCl, 5 mM MgCl_2_, 20% glycerol, 2.5 mM DTT, 2.5 mM EDTA pH 8, 250 μg/ml polydI-dC, 1 mg/ml BSA.).

For the SELEX binding reaction, 30 mL reactions were set up as follows: 9 μL of SELEX library diluted with water at final concentration of 200 nM, 15 μL of HES2 or ASCL2 wild type or mutant protein diluted with dialysis buffer at final concentration of 133.33 nM, and 6 microliters of 5 × binding buffer. Both control and SELEX binding reactions were assembled and incubated for 30 min at room temperature. The EMSA gel was pre-running during the binding reactions for the last 10 min. After the incubation, the pre-running of the gel was stopped, samples were loaded into the lanes, and the gel was run for approximately 100 min at 150 V (4°C). After the run stopped, the bound bands were cut out (Supplementary Fig. S11), purified, PCR amplified, and sequenced with NextSeq 500 according to protocol from Kribelbauer *et al.* [49].

### SELEX data analysis

ProBound [22] was applied for all eight protein samples to construct binding free energy models using the JSON configuration file in Supplemental Data SD1, resulting in symmetrical motif matrices with a CANNTG E-box core.

### Wild type and mutant ASCL2 EMSA experiment

EMSA assays were performed using ASCL2 wild type and mutant protein and ^32^P-labeled DNA probes. Protein samples were prepared using the same procedure as above. The DNA probes used, however, were different to avoid affinity towards the flanking regions:

CACGTG probe: TAGCCAATAACTTCGTCCCT-**CACG** **TG**-CATATAAGGAAGATCTAACCACCAATTTGG

CAGCTG probe: TAGCCAATAACTTCGTCCCT-**CAGC** **TG**-CATATAAGGAAGATCTAACCACCAATTTGG

Each probe was synthesized with ^32^P-labeled SRI sequence (CCAAATTGGTGGTTAGATCTTCC, reverse complementary to the end of the probe sequence) through annealing and Klenow reactions.

Around 50 nM of probe was combined with 500 nM, 1 μM, and 2 μM of protein, respectively, in each binding reaction, and the same reagents as used in Feng *et al.*, [50]. The mixture was loaded into a polyacrylamide gel after 30 min of reaction time. An EMSA gel was run for 100 min at 150 V (4°C), and dried and imaged using a phosphor-imaging plate and Typhoon gel imager.

The free energy difference associated with the sequence difference between CACGTG to CAGCTG of each ASCL2 wild-type or mutant sample was calculated by measuring the band intensities of the bound and unbound band using ImageJ and using the following equation:


\begin{eqnarray*}
\frac{{\Delta \Delta G}_{\mathrm{CG} \rightarrow \mathrm{GC}}}{RT} = \ln \left[ \left( \frac{ \mathrm{Bound}_{\mathrm{CG}} }{ \mathrm{Unbound}_{\mathrm{CG}}} \right) / \left( \frac{ \mathrm{Bound}_{\mathrm{GC}} }{ \mathrm{Unbound}_{\mathrm{GC}}} \right) \right]
\end{eqnarray*}

Here *Bound* and *Unbound* represent the measured band intensities of the corresponding lanes.

## Results

### A collection of SELEX-derived DNA binding specificity models for wild-type bHLH factors

bHLH proteins function as homo- and/or heterodimers that prefer to bind to sequences whose core matches the reverse-complement symmetric consensus pattern CANNTG (with “N” denoting any of the four nucleobases) called the enhancer box or E-box [51]. We collected *in vitro* DNA binding datasets for 147 human and mouse DNA binding domains from the bHLH family [16, 34, 35] and used ProBound [22] to obtain biophysically interpretable and accurate binding free energy models. Because bHLH homodimers are expected to have palindromic binding preferences (meaning that the binding free energy differences associated with base-pair substitutions are subject to reverse complement symmetry, but not that the binding sites themselves need to be palindromic), we configured ProBound to impose reverse-complement symmetry on its binding free energy parameters [22]. This yielded a binding model that matches the symmetrical CANNTG consensus E-box for 96 out of the 147 experiments analyzed, covering 54 distinct bHLH proteins. Further filtering for high intrinsic model quality resulted in a compendium of DNA recognition models for 52 bHLH factors (Supplemental Data SD2). For each TF included in this training set, the cloned sequence for each protein covers the 53-amino-acid bHLH HMMER profile, with a consensus Glu residue at position 9 [31]. With only a few exceptions, the preferred DNA binding site contains one of three E-box variants (CACGTG, CAGCTG, or CATATG). Protein sequences were aligned using the HMMER [52] profile of the bHLH family; alignment of the DNA binding specificity models was trivial thanks to their imposed palindromic symmetry. See **Materials and methods** for details.

### A reference-free and robust tetrahedron representation of DNA base preference

**Figure 1.**
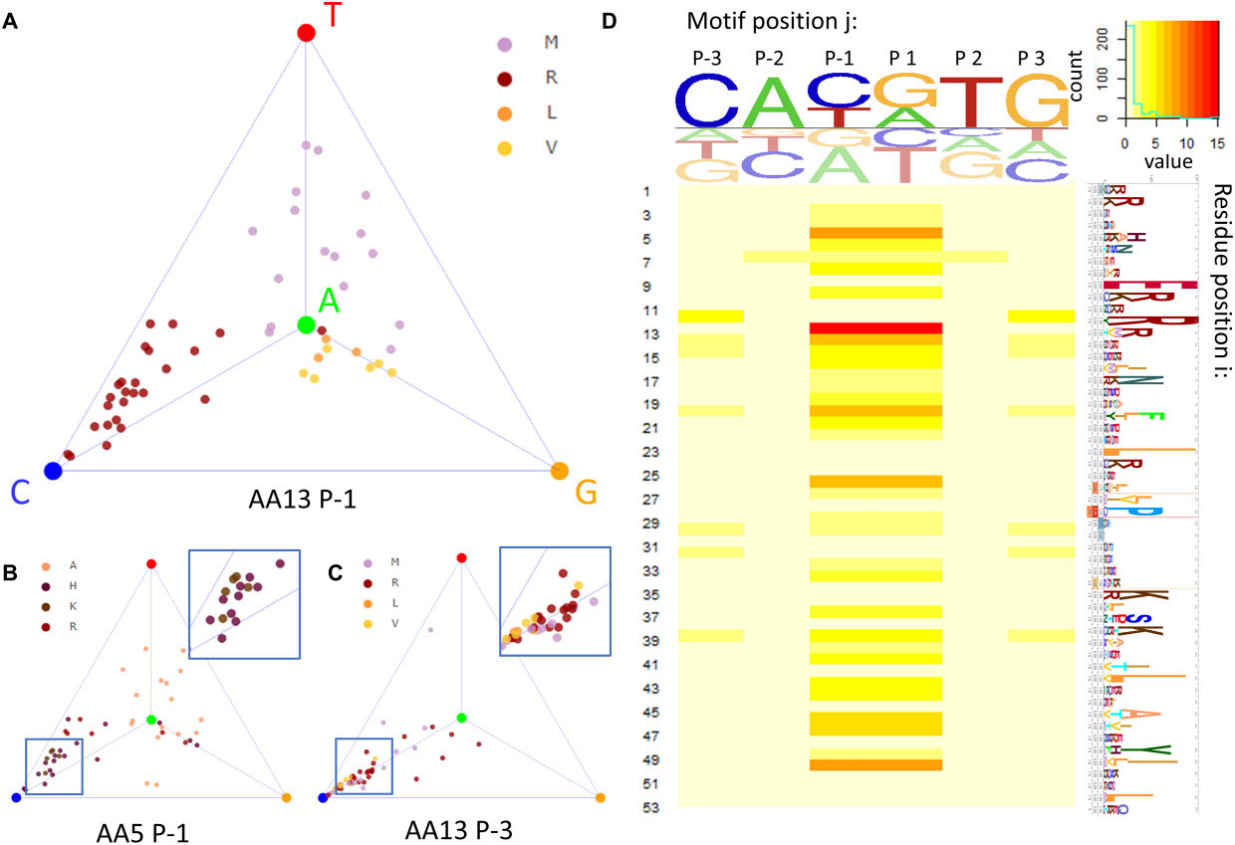
Family-level analysis of amino acid sequence determinants of DNA binding specificity for bHLH proteins. (**A**–**C**) Tetrahedron representation of base preference at DNA position −1 (panels A and B) or −3 (panel C). Each point represents a different bHLH factor and is colored according to the amino acid identity at protein position 13 (panels A and C) or 5 (panel B). To emphasize the association between amino acid residue identity and base preference, samples with Met, Arg, Leu, and Val at position 13 and samples with Ala, His, Lys, and Arg at position 5 are shown in the tetrahedron. (**D**) Heatmap showing the *P*-value from a MANOVA test between amino acid identity and position within the tetrahedron. Logo on top of the heatmap shows the average base preference of all bHLH sample collected. Logo to the right of the heatmap shows the HMMER alignment of bHLH samples collected [67].

The DNA binding specificity of each TF is defined in terms of a matrix of binding free energy differences ΔΔ*G* associated with base substitutions at each position in the DNA binding site. Setting ΔΔ*G* = 0 for the preferred base leaves three independent ΔΔ*G* parameters for each position in the DNA binding site. The corresponding relative affinity parameters are equal to exp(–ΔΔ*G*/*RT*) and together constitute a position specific affinity matrix (PSAM) [36]. When the goal is to explain variation in ΔΔ*G*/*RT* in response to variation in the amino acid sequence across set of TFs from the same family, the preferred base at a given position will not necessarily be the same for each TF. Moreover, for bases that are strongly disfavored at a given position, the corresponding ΔΔ*G*/*RT* values as estimated from the binding data can have large fluctuations that are not biophysically meaningful as they all correspond to a relative affinity near zero. These technical issues are addressed by mapping the ΔΔ*G*/*RT* values to a three-dimensional position within a tetrahedron whose vertices correspond to the four bases (Fig. 1A and Supplemental Data SD3; see **Methods** for details). Proximity of a particular TF to one of the vertices reflects a preference for the corresponding base.

### Mapping the protein sequence determinants of DNA binding specificity for bHLH factors

As a first step towards performing family-level analysis of how the DNA binding specificity of bHLH TFs is determined by their protein sequence, we focused on DNA position −1 in the PSAM for each TF and labeled/colored each corresponding point in the tetrahedron according to the amino acid identity at residue position 13 in the protein sequence alignment (Fig. 1A). Visual inspection suggests that there is a trend for TFs with an arginine at protein position 13 to prefer binding to DNA sequences with a cytosine at nucleotide position −1, while a methionine at the same protein position confers a preference for thymine. A similar plot can be generated for other combinations of DNA binding site position and protein alignment position. When a different residue position is chosen in the alignment of TF protein sequences, the coloring of the points changes (Fig. 1B); when a different position within the DNA binding site is chosen, the position of each point changes (Fig. 1C).

To systematically dissect the relationships between amino acid identity at residue positions in the bHLH protein sequence alignment and base preference at nucleotide positions within the DNA binding site in a way that also assesses statistical significance, we performed a series of multidimensional analysis of variance (MANOVA) tests. Each MANOVA considers the positions of points within the tetrahedron that represented the binding affinities at a specific motif position. The three-dimensional tetrahedral coordinate plays the role of dependent variable in these tests, while the (categorical) independent variable is the amino acid identity at a given position in the TF protein alignment. For each combination of protein and DNA position, the MANOVA yields a *P*-value that quantifies the statistical significance of the functional association. Because the binding model is reverse-complement symmetrical, the p-values for nucleotide positions −1, −2, and −3 are the same as +1, +2, and +3, respectively (Fig. 1D).

We found that of the 53 residue positions in the bHLH protein alignment, 9 are significantly associated with base preference at nucleotide position −1/+1 (*P* < 0.001 after Bonferroni correction [53]). Visualizing the p-values from the MANOVA test along the structure of PHO4 (Supplementary Fig. S3A) shows that, as expected, the residues in direct contact with the DNA major groove (e.g. positions 5, 13, 14, and 15) tend to have the most significant associations. Base preference at nucleotide position −1/+1 is greatly influenced by amino acid identity at residue position 13 (Supplementary Fig. S3B), consistent with the fact that in PHO4, the sidechain of Arg13 directly interacts with the base-pairs at position −1 and + 1 through hydrogen bonds that are known to be crucial for standard E-box preference [54, 55]. Available structures of bHLH–DNA complexes with other amino acids at residue position 13 also provide a mechanistic rationale for the observed difference in base preference (Supplementary Fig. S3C–E).

Our functional analysis suggests that amino acid variation at residue positions not in direct contact with DNA can also affect base preference. We analyzed structural data to find plausible mechanistic explanations for these cases. The residue at position 20 mediates a contact at the homodimerization interface. Having Phe or Met here promotes a preference for G and T at DNA position −1, possibly due to the larger side-chain size, while the smaller side chains of Ile and Leu confer a preference for C (Supplementary Fig. S4A, B). Structural data of protein–DNA complexes for PHO4 (PDB ID 1A0A) and Myod1 (PDB ID 1MDY) shows that for Phe20 and Leu20, the dimerization angle at the DNA binding residues is different between CACGTG and CAGCTG, with the distance between the C-alphas of Glu9 equal to 19.1 Å for CACGTG and 19.9 Å for CAGCTG [54, 56]. At position 50, also distant from the DNA, the positively charged Lys and Arg, as well as Gln, are associated with a preference for C at DNA position −1, likely due to a propensity to form an electrostatic and hydrogen bond with Glu, Gln, or Asp at residue position 22. The tighter interaction between the two monomers promotes narrower docking onto DNA, which may facilitate C recognition (Supplementary Fig. S4C, D). Among the non-DNA contacting residue positions, position 20 and 50 have not been previously associated with bHLH binding specificity, while positions 26, 46, and 47 still lack mechanistic explanations despite showing statistical significance in our analysis.

### A tetrahedron-based strategy for predicting DNA binding specificity from TF protein sequence

**Figure 2.**
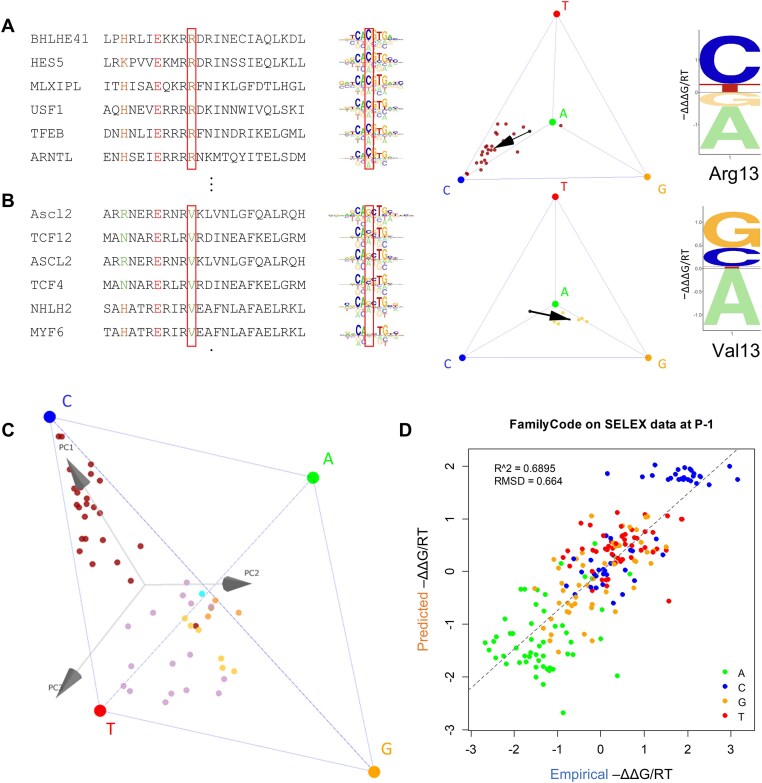
Predicting shifts in base preference associated with specific protein sequence features, and using PCA-regression to predict the DNA binding specificity of mutated bHLH proteins. (**A** and **B**) Protein alignment and binding motif of select bHLH proteins with either Arg (panel A) or Val (panel B) at residue position 13 (highlighted by red box). The energy logos and tetrahedron plots and the right show the base preference at DNA position –1 (red box). Arrows in the tetrahedrons indicate the shift relative to the overall centroid associated with the Arg13 and Val13 subset, which can again be represented by an energy logo (far right). (**C**) Principal component analysis of the set of tetrahedral coordinates defines a natural coordinate frame for each DNA position. (**D**) FamilyCode prediction performance on individually held-out wild-type bHLH proteins.

Beyond identifying positional correlations across the protein-DNA interface, the tetrahedron representation provides a natural starting point for predicting the effect of protein mutations on DNA binding specificity in a quantitative manner. For example, to estimate the effect of a point mutation from Arg to Val at residue position 13 in the bHLH domain on base preference at nucleotide position –1/+1, we can compute the centroid of all TFs with Arg13 or Val13, respectively (Fig. 2A and B), and then use the vector connecting the two centroids as a predictor of the change in binding specificity upon R13V mutation of any bHLH protein that contains an Arg at position 13.

To generalize this approach so that predictions can be made for any TF in the bHLH family, two issues need to be addressed. First, we would like to define a tetrahedral coordinate frame in a data-driven way, so that it optimally reflects the variation in chemical features that drives the interactions between amino acid side chains and the DNA ligand. To address this, we first perform principal component analysis (PCA) on the cloud of points within the tetrahedron (see **Materials and methods** for details). Second, we need to account for the fact that evolutionary selection has created dependencies between amino acid identities at distinct residue positions, which can confound the analysis when using multiple protein features simultaneously as a predictor of base preference. For instance, it would not be accurate to simply add up the individual effects of amino acid substitutions at two residue positions that are in perfect linkage disequilibrium with each other. To address this, we perform iterative feature selection using an F-test p-value threshold for each principal component (see **Materials and methods** for details).

Each of the three principal components (PC) defines a natural direction within the tetrahedron onto which the variation in base preference can be projected (Fig. 2C). Grouping TFs by amino acid identity at a given residue position again reveals interpretable patterns (Supplemental Data SD4). For instance, TFs containing protein feature Arg13 and Val13, respectively, are on opposite ends along PC1 (Fig. 2C). By performing a (one-dimensional) ANOVA for each individual principal component, functionally informative residue positions can be mapped (Supplemental Data SD5). The difference between the mean across all TFs and the mean of the subset that matches a specific protein sequence feature such as Arg13 reflects the quantitative shift in base preference associated with that feature. Whenever a particular amino acid in the predicted TF is not present in the training data, we estimate its centroid as the mean of the centroids of all other amino acids at the same position, weighted by amino acid substitution distance according to BLOSUM62 [40].

### Cross-validation of specificity predictions on held-out wild-type bHLH factors

As an initial assessment of this prediction scheme, which we refer to as FamilyCode, we performed leave-one-out cross-validation across the bHLH family, in which we built a PCA-regression model from all TFs except one, and then used this model to predict the tetrahedral position of the held-out TF. The prediction accuracy was 0.69 in terms of coefficient of determination (*R*^2^) and 0.66 in terms of root-mean-squared deviation (RMSD) of ΔΔ*G*/*RT* (Fig. 2D). For reference, we also computed these metrics of similarity between technical replicates across a subset of 20 bHLH factors for which we had access to two HT-SELEX replicates (*R*^2^ = 0.72, RMSD = 0.54, Supplementary Fig. S5A). We compared with a previously proposed strategy that simply takes the DNA binding specificity of the closest-paralog as the prediction [57], as well as with a refinement of this approach called similarity regression [39]. Our PCA-regression model outperforms both schemes in predicting for the DNA binding free energies at the −1/+1 position by a significant margin (closest-paralog: *R*^2^ = 0.63, RMSD = 0.75, *P*-value < 10^–24^, bootstrap t-test; similarity regression using pretrained weights: *R*^2^ = 0.58, RMSD = 0.79, *P*-value < 10^–33^, bootstrap *t*-test). See Supplementary Fig. S5B–D for details. Note that unlike our feature-based approach, neither of the alternative prediction methods provides detailed information about dependencies between specific DNA positions and specific TF protein positions. To explore the effects of “information leakage” between the held-out TF on the one hand, and TFs with closely similar proteins sequences in the training set on the other, we also implemented a cross-validation procedure in which we excluded from the training set those TFs within a given hamming distance from the test TF. We found that the prediction accuracy of our FamilyCode consistently exceeded that of sequence homology and similarity regression at varying thresholds, in further evidence of its ability to generalize (Supplementary Fig. S6).

### Experimental validation of specificity shift predictions for mutant bHLH factors

Since high-throughput *in vitro* binding data are available for most human wild-type TFs [16, 34, 35], a more relevant application of FamilyCode is to predict whether and how DNA binding specificity is modulated by a particular mutation in the TF protein sequence. The number of natural TF variants in the human population is far too large for an experimental approach to be practical in that case.

**Figure 3.**
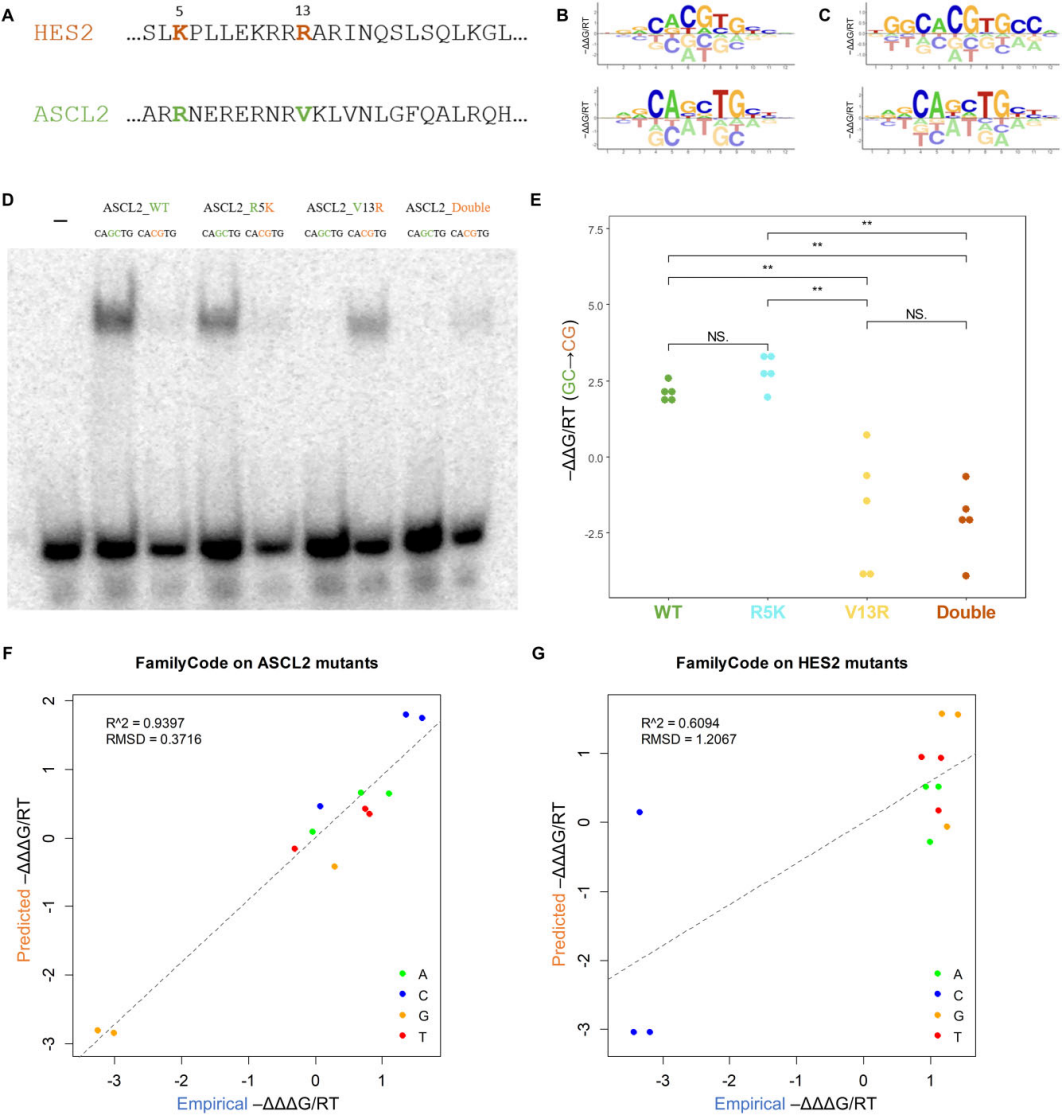
Validating FamilyCode predictions for mutant bHLH proteins HES2 and ASCL2. (**A**) Sequences of wild-type human bHLH factors HES2 and ASCL2, with mutated positions 5 and 13 indicated. (**B**) Binding energy logos inferred from HT-SELEX data for wild-type HES2 and ASCL2 using ProBound. (**C**) Binding energy logos inferred from SELEX-seq experiments for wild-type HES2 and ASCL2 using ProBound. (**D**) EMSA assays showing the binding preference between DNA probes CACGTG and CAGCTG, respectively, for both wild-type and mutant ASCL2 protein. (**E**) Statistical analysis of the changes in EMSA gel band intensity in panel D. (**F**) Predicted versus experimental values of ΔΔΔ*G* for HES2 mutants K5R, R13V, and K5R + R13V. (**G**) Same for ASCL2 mutants R5K, V13R, and R5K + V13R.

We wished to perform experimental validation of FamilyCode using two different bHLH proteins: HES2, which energetically prefers the standard E-box CACGTG (cytosine at position −1), and ASCL2, which prefers the E-box variant CAGCTG. Among other differences, wild-type HES2 contains Lys5 and Arg13 whereas wild-type ASCL2 has Arg5 and Val13 (Fig. 3A). We first performed SELEX-seq on wild-type HES2 and ASCL2 (see **Materials and methods**) and used *ProBound* to infer binding models (Fig. 3C). Comparing with wild-type models inferred from existing HT-SELEX data for the same TFs (Fig. 3B), we found that while the ΔΔ*G*/*RT* coefficients for ASCL2 were very similar (RMSD = 0.32), those for HES2 were less reproducible (RMSD = 0.61). This discrepancy is likely due to variation in protein solubility (only ASCL2 was tagged with mScarlet) and/or absolute binding affinity. The lower consistency among wild-type replicates for HES2 pointed to ASCL2 as being the more suitable TF for assessing the accuracy of our predictions.

We first used FamilyCode to predict the base preferences at position −1/+1 for ASCL2 proteins carrying the mutations R5K and/or V13R. In making these predictions, we treated each mutant as an unseen protein and predicted its binding specificity (in terms of ΔΔ*G*/*RT*) the same way as for held-out wild-type TFs above. To experimentally validate these predictions, we performed electrophoretic mobility shift assays (EMSAs) with wild-type and mutant ASCL2 protein (see **Methods**). Consistent with our predictions, these assays showed that the V13R single mutant and the R5K + V13R double mutant of ASCL2 had higher affinity for the canonical E-box (CACGTG) than the alternative E-box (CAGCTG) preferred by wild-type ASCL2 and its R5K mutant (Fig. 3D and E).

When considering a missense mutation in the protein sequence of a TF, the effect on base preference at a given DNA position is defined by a set of ΔΔΔ*G*/*RT* values that quantify the *shift* in ΔΔ*G*/*RT* values from the reference TF to the mutant TF (Supplementary Fig. S2). Here again, we can control the undue influence of large fluctuations in the estimated value of unfavorable bases by using the tetrahedral representation to estimate ΔΔΔ*G*/*RT*, rather than directly subtracting the ΔΔ*G*/*RT* values for the mutant and reference from each other (see **Methods**).

We retrained our FamilyCode model on all available wild-type bHLH data while only fitting amino acid substitution coefficients for the 5^th^ and 13^th^ protein positions. Predictions for the HES2 and ASCL2 mutants were made in terms of ΔΔΔ*G*/*RT* values at DNA positions −1 and + 1. We performed additional SELEX-seq assays for the four single mutants HES2 (K5R or R13V) and ASCL2 (R5K or V13R) and two double mutants HES2 (K5R + R13V) and ASCL2 (R5K + V13R), and applied *ProBound* to obtain a binding model for each variant. Empirical values for ΔΔΔ*G*/*RT* were derived using a procedure based on the tetrahedron embedding, which is less sensitive to the larger error in binding free energy value for disfavored bases (see **Methods**). High prediction accuracy for the variant effects was observed for ASCL2 mutants (Fig. 3F; *R*^2^ = 0.94 and RMSD = 0.37). The lower prediction accuracy for HES2 mutants (Fig. 3G; *R*^2^ = 0.61 and RMSD = 1.21) is consistent with the lower reproducibility of the wild-type HES2 model noted above.

### Extension of our approach to wild-type and mutant bHLH data from the PBM platform

Our family-level prediction model for the bHLH family so far relied on DNA binding specificity models for individual TFs inferred from count-based high-throughput SELEX data. To leverage the large existing body of fluorescence-based protein binding microarray (PBM) data [13], we can also directly fit ΔΔ*G*/*RT* parameters to raw PBM data via a non-linear biophysical model, as originally implemented by the MatrixREDUCE [21] and FeatureREDUCE [30] algorithms. Specifically, we extended the functionality of our recent *PyProBound* [43] implementation of *ProBound* [23] from a Poisson-based to a Gaussian-based maximum-likelihood model to allow direct analysis of PBM probe fluorescent intensities (see **Materials and methods**). This allowed us to repeat the family-level analysis for the bHLH family using data from a distinct experimental platform and for a different set of individual TFs (most of the SELEX-based bHLH models in MotifCentral are for human TFs; most of the PBM data for bHLH factors available through CisBP are for *C. elegans* factors).

We obtained normalized PBM data for 88 bHLH domains from CisBP [33, 57, 58], constructed sequence-to-affinity models for each TF using PyProBound while imposing palindromic symmetry, and aligned protein sequences to the PFAM model of bHLH. For 80 of these bHLH domains, at least two replicate datasets available, which allowed us to determine the baseline reproducibility (*R*^2^ = 0.55, RMSD = 1.52, Supplementary Fig. S7A). Applying the same MANOVA approach as above to map dependencies between protein positions and DNA positions, we found that the most significant associations are reproducible (Supplementary Fig. S8A and B). Next, we built a FamilyCode model and performed leave-one-out cross-validation of prediction of wild-type bHLH factors. Prediction performance for DNA position −1/+1 (*R*^2^ = 0.63, RMSD = 1.39) was better than that of the closest-paralog approach (*R*^2^ = 0.57, RMSD = 1.53), and similar as that of similarity regression (*R*^2^ = 0.62, RMSD = 1.45); see Supplementary Fig. S7B-D for details.

**Figure 4.**
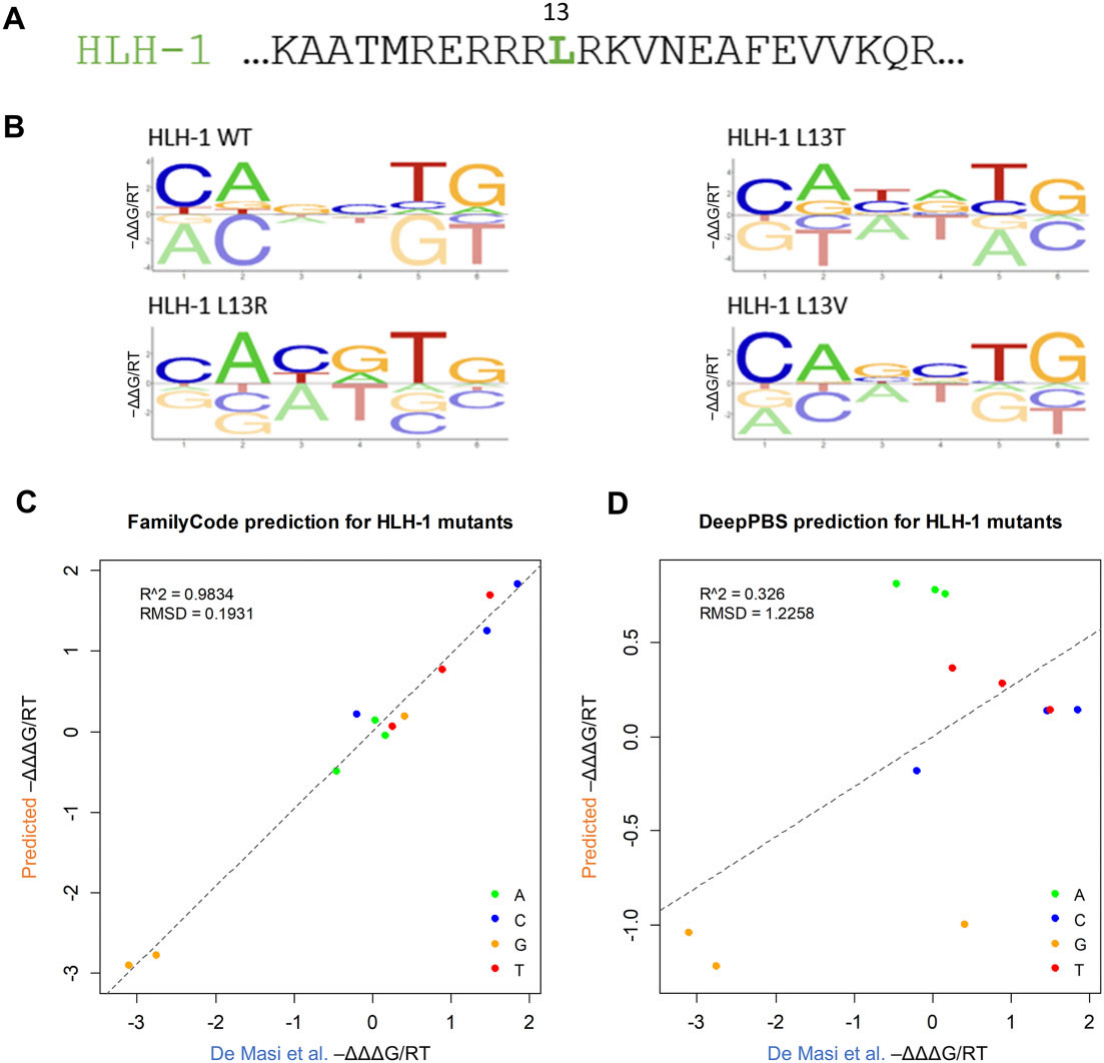
Validating family-level predictions for mutant HLH-1 protein. (**A**) Wild-type sequence of *C. elegans* factor HLH-1, with mutated position 13 indicated. (**B**) Energy logos for wild-type and mutant HLH-1 factors inferred from PBM data using PyProBound. (**C**) Predicted versus empirically values of ΔΔΔ*G* for the three HLH-1 mutants, with predictions made using FamilyCode (this work). (**D**) The same comparison, with predictions made using the DeepPBS method of Ref. [45].

For a more rigorous validation of our ability to predict DNA binding specificity effects for unseen bHLH variants, we used PBM data for three unseen mutants of HLH-1 collected by De Masi *et al.* [33]. Wild-type HLH-1 has Leu at bHLH domain residue position 13 and preferably binds to the CAGCTG E-box (Fig. 4A and B); note that among the two available replicates for wild-type HLH-1 in CisBP, we chose the one with this same half-site preference (CAG). We predicted base preference shifts associated with the HLH-1 mutations L13R, L13T, and L13V (in the form of ΔΔΔ*G*/*RT* values) from binding specificity models for wild-type bHLH factors alone using the same procedure used for HES2 and ASCL2 above, and compared these directly with empirical ΔΔΔ*G*/*RT* values derived from the PBM data for the HLH-1 mutants (Fig. 3F and G). Our prediction accuracy for motif position –1 was excellent (*R*^2^ = 0.98, RMSD = 0.19; see Fig. 4C), and compared favorably to that of a state-of-the-art structure-based deep learning algorithm named DeepPBS [45] (*R*^2^ = 0.33, RMSD = 1.23; see Fig. 4D).

### Mapping DNA binding specificity determinants for homeodomains

To demonstrate the generalizability of our FamilyCode framework, we applied the same workflow to the homeodomain (HD) family of TFs. To obtain a more comprehensive training set, we used PyProBound to infer sequence-to-affinity models from all available HT-SELEX and PBM data for HDs [16, 34, 35, 51, 57]. For HT-SELEX, a total of 259 unique HDs sourced from MotifCentral [22] had at least two replicates. For PBM, a total of 314 unique HDs are available in CisBP, of which 272 have a corresponding Uniprot entry. The final set of sequence-to-affinity models covered 414 unique HDs (Supplemental Data SD6).

**Figure 5.**
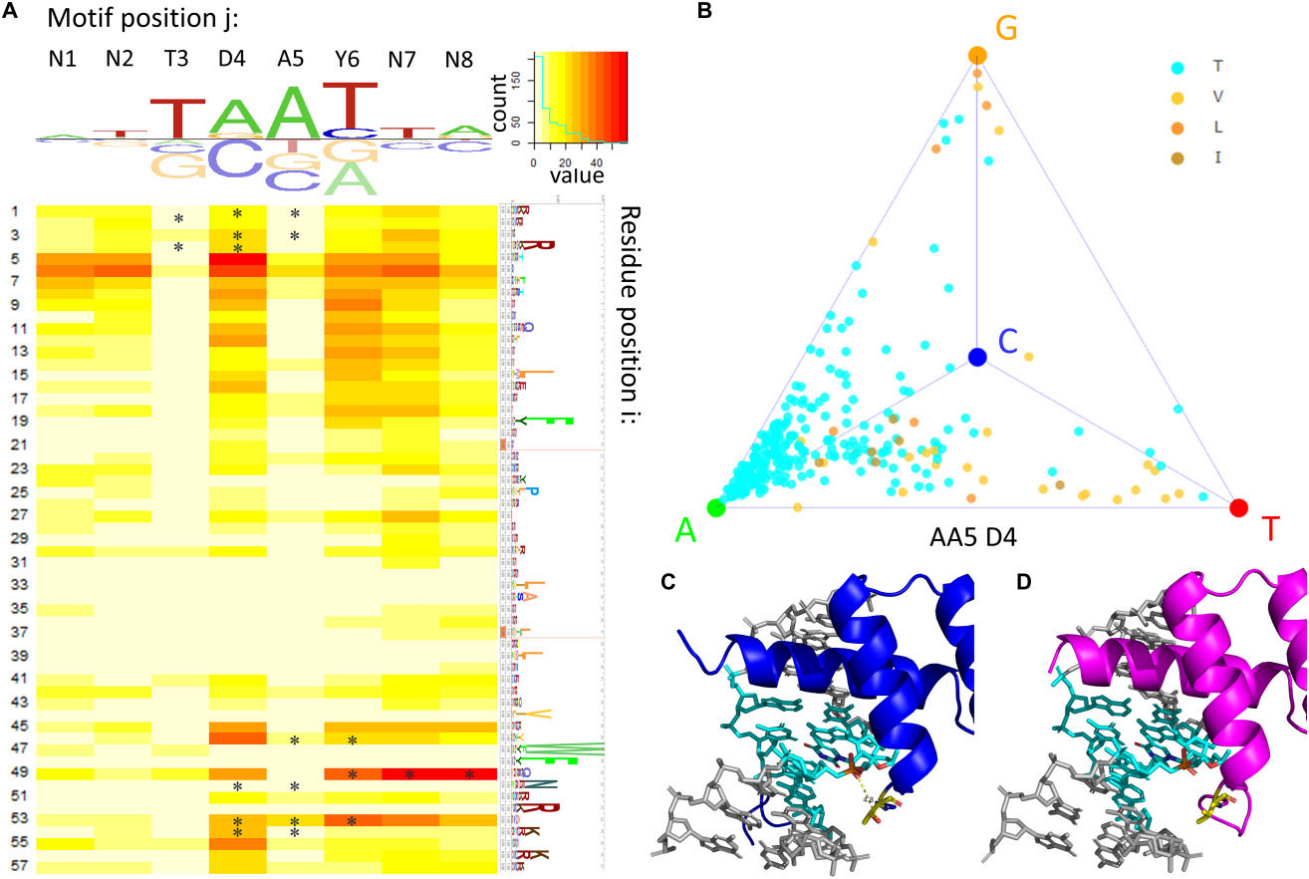
Mapping specificity determinants for homeodomain (HD) proteins using a model trained on HT-SELEX and PBM data. (**A**) Heatmap showing the *P*-value from a MANOVA test between amino acid identity and position within the tetrahedron. Asterisks mark base contacting residues. The logo on top of the heatmap shows the average base preference of all HD samples collected. Logo to the right of the heatmap shows the HMMER alignment of bHLH samples collected [67]. (**B**) Tetrahedron representation of base preference at DNA position D4, with TFs colored according to amino acid identity at residue position 5. Only TF sample with amino acid residue identities Thr, Leu, Ilu, and Val are shown in the tetrahedron. (**C** and **D**) Structural inspection of the interaction between AA5 and D4 on Scr (**C**) and CDX1 (**D**), using PDB ID 2R5Z [60] and 7Q3O. Figures created using 3DNA [68].

Tetrahedral MANOVA analysis for this multi-platform compendium of HD binding models (Fig. 5A and Supplementary Fig. S8C and D) identified several residue positions at which variation in amino acid identity had been previously shown to impact DNA binding preference [26, 29]. However, while most of these previous methods used structural information as part of their analysis, our MANOVA analysis is structure-agnostic.

Our finding that for HD positions 49 and 53 there is a strong association with base preference at DNA positions Y6 and N8 is consistent with previous findings [24, 26, 27, 44, 59], as is the finding that HD position 46 is associated with base preference at DNA position D4 [26, 27, 44]. DNA-contacting HD positions 4 and 54 were not found to be associated with DNA binding specificity variation in our analysis due to their high conservation.

HD position 5 was previously identified as a highly associated residue in the prediction scheme by Christensen *et al.* [27], but was overlooked by structure-based approaches as residue 5 does not directly contact DNA [27]. This position is highly associated with the base recognition at the D4 position of the motif, with Thr5 showing a consistent preference towards an A, while the hydrophobic residues Leu5, Ile5, and Val5 prefer either a G or a T (Fig. 5B, Supplemental Data SD7). For example, comparing the structures of Scr and CDX1, which have a Thr5 and Leu5 respectively, indicates that the side chain of AA5 can either form a hydrogen bond with the DNA backbone or not, depending on the amino acid identity (Fig. 5C and D) [60]. Christensen *et al.* and our method predict the same set of four HD positions as top determinants of DNA binding preference, which is satisfying because their data were from a different experiment platform (bacterial one-hybrid, or B1H). However, we note that our MANOVA approach is the only one that assesses the statistical significance of the identified positional associations.

### Predicting the effect of missense mutations in homeodomains on DNA binding specificity

A recent study [29] used PBM technology to profile how 92 different mutations in 30 HDs altered their DNA binding specificity, allowing us to directly and quantitatively assess our ability to predict the impact of mutations using our model as trained on wild-type HDs alone. To this end, we first used PyProBound to infer ΔΔ*G*/*RT* models from the raw PBM intensities; this was done separately for each wild-type or mutant HD profiled in [29]. In each case, we used the fraction of the variance in the raw PBM intensities explained by our model fit to select the highest-quality replicate (see **Materials and methods**). Subsequently, we used our tetrahedral representation to robustly estimate empirical ΔΔΔ*G*/*RT* values for each HD mutant.

**Figure 6.**
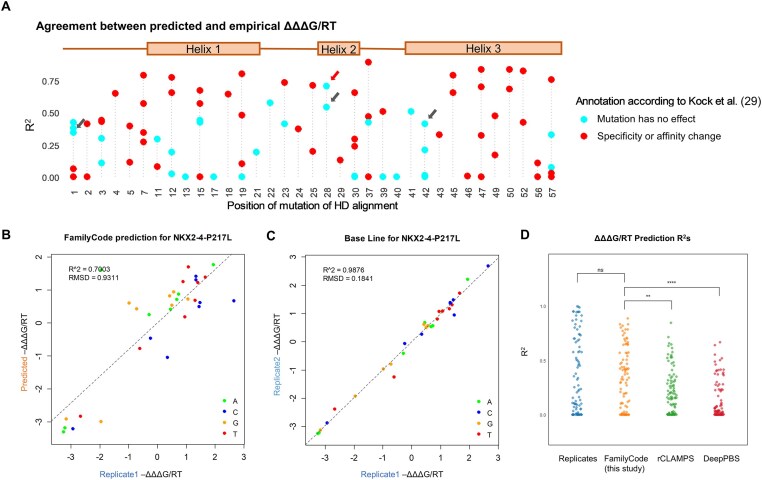
Validation of family-level predictions for disease-associated homeodomain mutants. (**A**) Predictive performance of FamilyCode as trained on wild-type homeodomains. Shown is the correlation (R^2^) between predicted and empirical ΔΔΔ*G*/*RT* values for each of the 92 mutants profiled by Kock *et al.* [29]. Arrows denote mutants for which we observed consistent effects on DNA binding specificity that have not previously been reported. (**B**) Replicate-to-replicate comparison of empirical ΔΔΔG/RT values for the P217L mutant of NKX2-4, indicated by the red arrow in panel A. (**C**) FamilyCode prediction versus empirical ΔΔΔ*G*/*RT* values for the same mutant. (**D**) Comparison of predictive performance of FamilyCode with rCLAMPS [44] and DeepPBS [45]. Significance levels were computed using the paired Wilcoxon signed-rank test (ns: not significant; * *P* < 0.05; ** *P* < 0.01; *** *P* < 0.001; **** *P* < 0.0001).

Supplementary Fig. S9A shows the empirical effect size the mutation in terms of the magnitude of the ΔΔΔ*G*/*RT* range (difference between largest and smallest value) across all eight DNA positions, separately for each of the 92 mutants. There is a clear trend for mutants that were classified as having no effect on DNA binding by Kock *et al.* [29] to have a smaller impact according to our ΔΔΔ*G* range metric (*P*= 0.0024, Hypergeometric test with threshold of ΔΔΔ*G*/*RT* = 1). An overview of the correlation in empirical ΔΔΔ*G*/*RT* values between replicates is shown in Supplementary Fig. S9B, and examples for specific mutants are shown in Fig. 6B and C and Supplementary Fig. S10.

Fig. 6A shows how well the empirical ΔΔΔ*G*/*RT* values for the 92 HD mutants can be predicted by FamilyCode when trained on wild-type HD data alone. Supplementary Fig. S9 provides more information about the range and reproducibility of the empirical ΔΔΔ*G*/*RT* values. Four mutants (indicated by grey arrows in Fig. 6A and Supplementary Fig. S9) that were classified by Kock *et al.* as “no change” stood out in our own analysis as having a large empirical ΔΔΔ*G*/*RT* range, excellent empirical ΔΔΔ*G*/*RT* reproducibility, and good predictive performance of FamilyCode (Fig. 6A, Supplementary Fig. S9, and Supplementary Fig. S10). This reanalysis of the same raw PBM data illustrates how the combination of PyProBound and our tetrahedron-based method for estimating ΔΔΔ*G*/*RT* values allows for sensitive detection of mutant effects on DNA binding specificity.

The performance of FamilyCode (*R*^2^ for predicted v. empirical ΔΔΔ*G*/*RT* values) across all 92 HD mutants is shown in orange in Fig. 6D. It approaches the reproducibility in empirical ΔΔΔ*G*/*RT* values inferred from two replicates for the same mutant HDs shown in blue. Some of the mutants have a small *R*^2^ value in the empirical replicate comparison, which may simply indicate that the missense mutation has a small effect on base preference (${\mathrm{\Delta \Delta \Delta }}G/RT \ll$1). In general, however, our FamilyCode showed superior predictive power on the missense mutants compared to the state-of-the-art rCLAMPS model [44] for predicting HD specificity (*P* = 0.0057, paired rank sum test). The structure-based predictor DeepPBS [45] performed only modestly compared to both our FamilyCode method and rCLAMPS when applied to the same mutant HD validation set (Fig. 6D). Note that to ensure a fair comparison, we used the same tetrahedral transformation to compute ΔΔΔ*G*/*RT* values in all cases (see **Materials and methods**). It was previously shown that another structure-based prediction algorithm named ModCRE [61] is outperformed by rCLAMPS on the PWM prediction of TFs from the HD family. It is perhaps not surprising that family-specific prediction strategies such as FamilyCode and rCLAMPS outperform cross-family ones such as DeepPBS and ModCRE on family-specific prediction tasks.

## Discussion

In this study, we introduced and validated a modeling framework for predicting the quantitative impact of missense mutations on the DNA binding specificity of transcription factors. Amino acid substitutions can lead to loss of function related to a disruption of the TF’s ability to bind DNA, but our aim here was to predict more subtle quantitative effects on DNA binding preference, as these in turn might explain phenotypic effects associated with common or rare variants.

Each family of transcription factors is considered separately in our approach. While we focused on the bHLH and homeodomain families as a proof of concept, the approach is general, and could also be applied to other TF families that have a sufficiently larger number of members for which high-throughput DNA binding data are available. We exploit the fact that amino acid identity will vary at many positions in the protein alignment of wild-type transcription factors from a given family. Our family-level model estimates the average effect of amino acid substitutions at each position using high-throughput *in vitro* binding data for each TF in the training set. We want to stress that our validation was focused on the effect of single-residue substitutions in the context of natural genetic variation. While our family-level models can also be used to make predictions for TFs that are many substitutions away from a wild-type reference, we expect that the accuracy of the predictions would be significantly lower in those cases. Therefore, our approach may be less suitable for the *de novo* engineering of novel TFs with prescribed DNA binding preferences.

An important determinant of the quality of our predictions is simply the total number of TFs in the training set, which was several dozen in our study. For larger families, a collection of human TF paralogs may suffice, but TFs from multiple species can be naturally combined in our approach. In fact, since the tetrahedral representation of base preference that we use as the basis for our family-level analysis is completely general, high-throughput binding data from multiple platforms (e.g. SELEX and PBM) can be seamlessly integrated in a single model, as they all reflect the intrinsic DNA binding specificity of the TF.

As we have shown in previous work [22], high-throughput *in vivo* binding data such as ChIP-seq can yield models that are almost indistinguishable from those inferred from *in vitro* binding data as long as peak-agnostic analysis methods are used to infer the binding free energy coefficients. This implies that large-scale ChIP-seq data sets from projects such as ENCODE [62] might also be leveraged in the future to increase the number of TFs in the training set for family-level modeling, or to provide information about shifts in base preference associated with disease mutants. The downside of inferring binding free energy parameters from ChIP-seq data, however, is that they may be confounded by the effects of DNA methylation, chromatin accessibility, and/or cooperative interactions with co-factors. For this reason, we did not include any ChIP-seq derived models in our analyses.

We want to stress that while the latest generation of structure prediction algorithms [46] are now capable of predicting the 3D structure of a protein–DNA complex from its primary sequence to a reasonable degree, this is still a vastly simpler task than predicting the energetic effect of base-pair or amino acid substitutions at the protein-DNA interface. For the latter, there is currently no alternative to the combination of high-throughput experimental binding data combined with interpretable machine learning that we employed in this study. That said, there may still be a role for computational methods that predict the structure of the protein backbone: An implicit assumption in our family-level modeling is that the backbone geometry of the protein-DNA interface is fixed, and that the only thing that varies is the chemical identity of the base pairs and amino acid sidechains. However, some TF families such as basic leucine zipper (bZIP) factors are capable of binding to DNA in alternative homodimer configurations [30, 63], and leveraging structure prediction methods to account for this in the context of family-level modeling may be a valuable extension of this approach.

Our approach is structure-agnostic in that it aims to directly predict TF *function* (i.e. base preference at a particular DNA position) from TF *sequence* (i.e. the amino acid identity at each position in the protein alignment). The limited structural data that we presented only serves to illustrate that our functional analysis yields result that make mechanistic sense. The protein sequence alignment only serves to link corresponding residue positions across TFs. We do not use knowledge about the evolutionary relationships between the various TFs in the alignment, although these do lead to correlations between the binary amino acid indicators that we use as independent variables in our family model, which is one of the reasons why we use forward feature selection. It is conceivable that foundation models built from large sets of homologous protein sequences [64] can be leveraged to create a more informative mapping of individual TF protein sequences to a vector of continuous feature weights, thereby increasing the predictive performance of our family-level models. Finally, it has been demonstrated empirically that an equivalent missense substitution can have different effects on DNA binding in different HD mutants [29], and multi-domain zinc finger (ZF) transcription factors are known to exhibit strong dependencies between adjacent ZF domains [65, 66]. It would be interesting to leverage the tetrahedron representation to account for such dependence on protein sequence context when predicting mutation effects based on wild-type training data.

## Supplementary Material

gkaf831_Supplemental_Files

## Data Availability

All computer code developed for and used in this study, including scripts to generate all figure panels, is available at github.com/BussemakerLab/FamilyCode and doi.org/10.5281/zenodo.15858756. Raw sequencing data for the SELEX-seq assays we performed in this study is available in the NCBI Sequence Read Archive (SRA) via BioProject identifier PRJNA1244358.
