# Supplementary material for: Predicting the DNA binding specificity of transcription factor mutants using family-level biophysically interpretable machine learning
Source: Nucleic Acids Res. 2025 Aug 28;53(16):gkaf831. doi: 10.1093/nar/gkaf831 (PMC12392098; doi:10.1093/nar/gkaf831)
Supplement: gkaf831_Supplemental_Files [file gkaf831_supplemental_files.zip › Supplement.pdf]

SUPPLEMENT

Predicting the DNA binding specificity of transcription factor mutants using family-level biophysically interpretable machine learning

Shaoxun Liu[1], Pilar Gomez-Alcala[1], Christ Leemans[1], William J. Glassford[2], Lucas A.N. Melo[1], Xiang-Jun Lu[1], Richard S. Mann[2,3,+], Harmen J. Bussemaker[1,3,+]

[1]Department of Biological Sciences, Columbia University, New York, NY, USA.
[2]Department of Biochemistry and Molecular Biophysics, Columbia University, New York, NY, USA
[3]Department of Systems Biology, Columbia University, New York, NY, USA
[+]Corresponding authors (hjb2004@columbia.edu; rsm10@columbia.edu)

CONTENTS

List of Supplemental Data
Figures S1 through S11

LIST OF SUPPLEMENTAL DATA

SD1: JSON configuration file used for all ProBound analyses.

SD2: DNA recognition models for the 52 bHLH factors analyzed in this study.

SD3: Interactive 3D representations of tetrahedrons for bHLH examles. Open HTML files in browser to view and manipulate. Related to Figure 1A-C.

SD4: Empirical cumulative distribution of tetrahedral position along each principal component direction for various amino-acid positions. Related to Figure 3B.

SD5: Statistical significance of ANOVA test of PCs at DNA position –1/+1, –2/+2 and –3/+3.

SD6: DNA recognition models for the 414 HD factors analyzed in this study.

SD7: Interactive 3D representations of tetrahedrons for HD examples. Open HTML files in browser to view and manipulate. Related to Figure 5B.

All supplementary data files are available for download at bussemakerlab.org/papers/FamilyCode/.

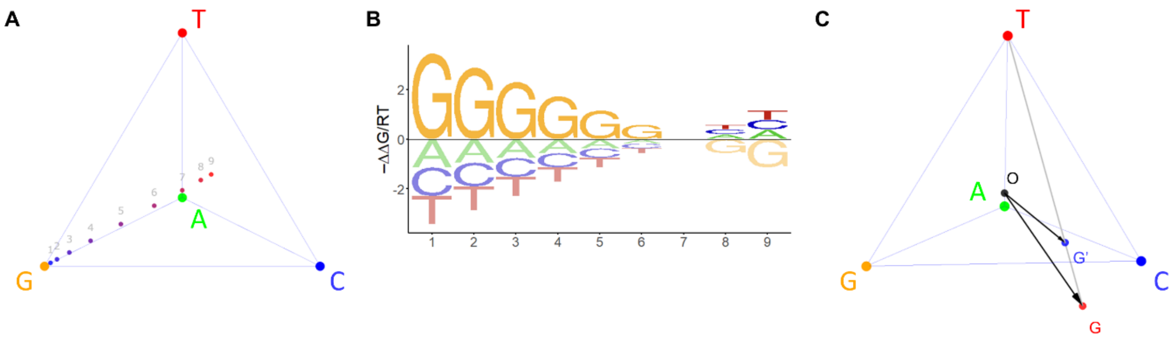

<u>Figure S1</u>: Illustration of the relationship between ΔΔG/RT and tetrahedral position. (**A**) Tetrahedron representation of base preference variation along a line between the G vertex and the center of the other three vertices, which also passes through the center of the tetrahedron. (**B**) Free energy parameters ($\Delta\Delta G/RT$) representing the base preference for the series of points in panel A. Point #7 corresponds to the center of the tetrahedron. (**C**) Illustration of the regulation procedure we used to enable the inverse mapping to free energy shifts ($\Delta\Delta\Delta G/RT$) to points outside the tetrahedron. Point O depicts the center of the tetrahedron where the preference towards all 4 bases is equal. For point G outside of the tetrahedron, the regularization procedure moves it along the line between G and the base it least prefers (T) to intersect with the plain that represents 0.01 relative affinity towards T, represented by G′.

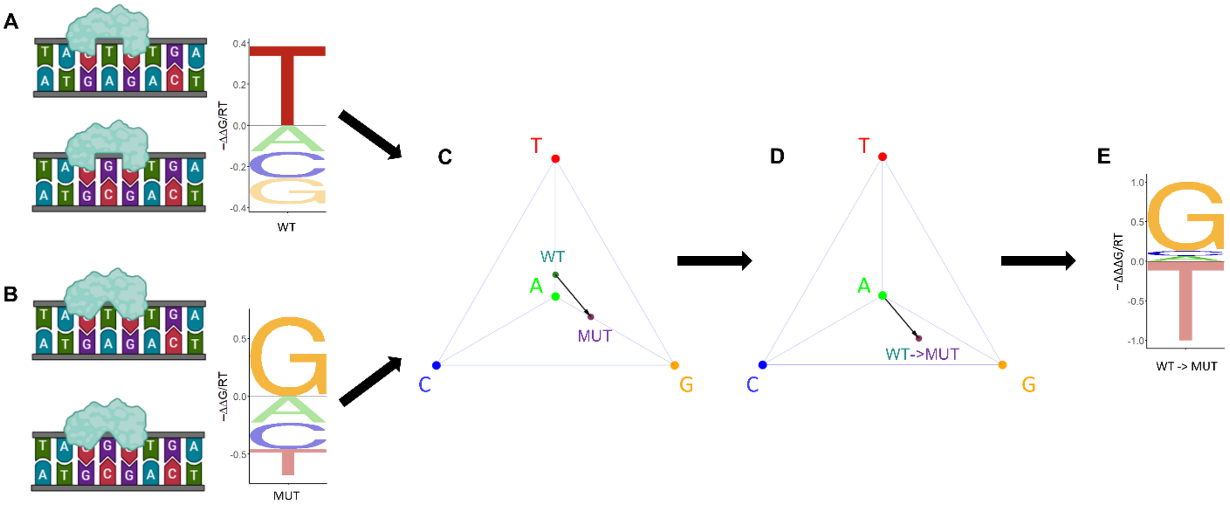

<u>Figure S2</u>: Illustration of tetrahedron-based procedure for calculating $\Delta\Delta\Delta G/RT$ values. (A) Example of a wild-type TF that prefers to bind with T over G. (B) Example of a mutant of the TF shown in panel A. The mutant TF prefers base G over T. (C) Representing the shift in base preference by mapping the binding free energy parameters $\Delta\Delta G/RT$ for the wild-type and mutant TF, respectively, to points within the tetrahedron. (D) Representing the shift in base preference as a vector in tetrahedron space. (E) Inverse mapping of the tetrahedral shift to a shift $\Delta\Delta\Delta G/RT$ in binding free energy parameters. (Panels A and B were created using BioRender.)

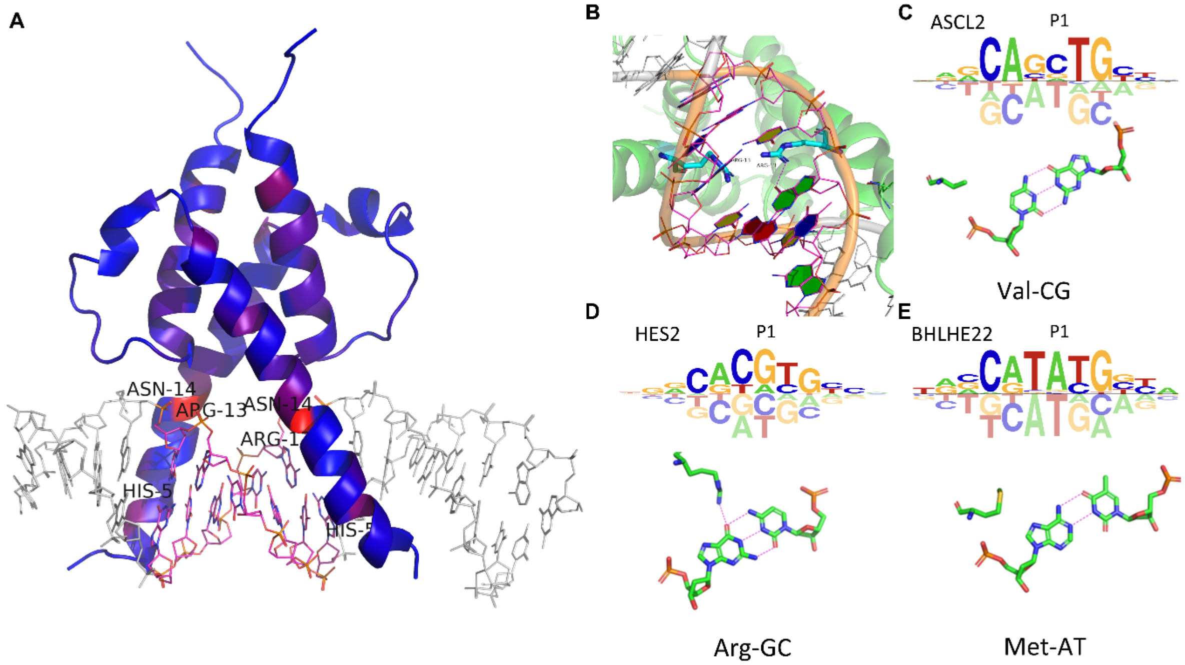

<u>Figure S3</u>: Structural analysis of bHLH DNA-binding residues. (**A**) Statistical significance at position –1 shown in the context of the co-crystal structure for Pho4 (red denotes significant p-values). (**B**) Detailed view of the interaction between Arg13 and a GC base pair at motif position +1. The Arg13 sidechain is colored in cyan. The E-box is shown in magenta using DNA blocks. (**C**-**E**) Additional examples of interactions between amino-acids and base pairs at residue position 13 and DNA position +1. Structural images generated using 3DNA/DSSR (x3dna.org)(68). Hydrogen bonds are shown as pink dashed lines.

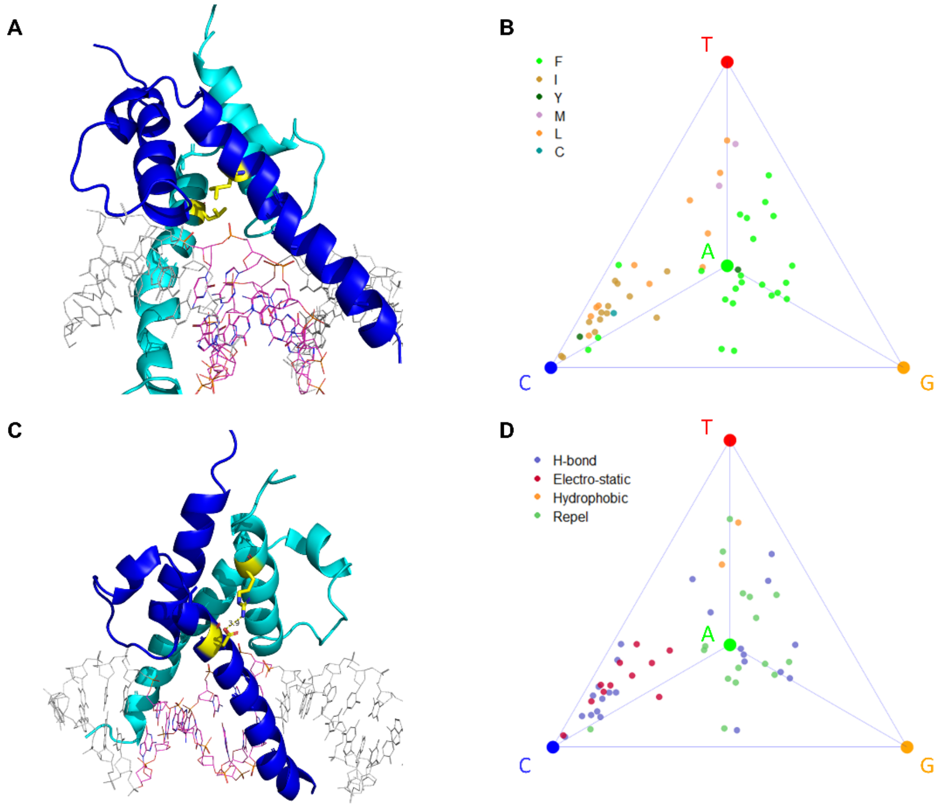

<u>Figure S4</u>: Associations found for residue positions 20 and 50 in the bHLH family analysis. (**A**) Structure of bHLH protein PHO4 with residue position 20 highlighted in yellow, and blue and cyan indicate the two bHLH monomer subunits. (**B**) Tetrahedron representation for DNA position –1, with coloring according to the amino acid identity at residue position 20. (**C**) Same as panel A, but for residue positions 22 and 50. (**D**) Tetrahedron representation of DNA position –1 with coloring according to the type of contact between residue positions 22 and 50.

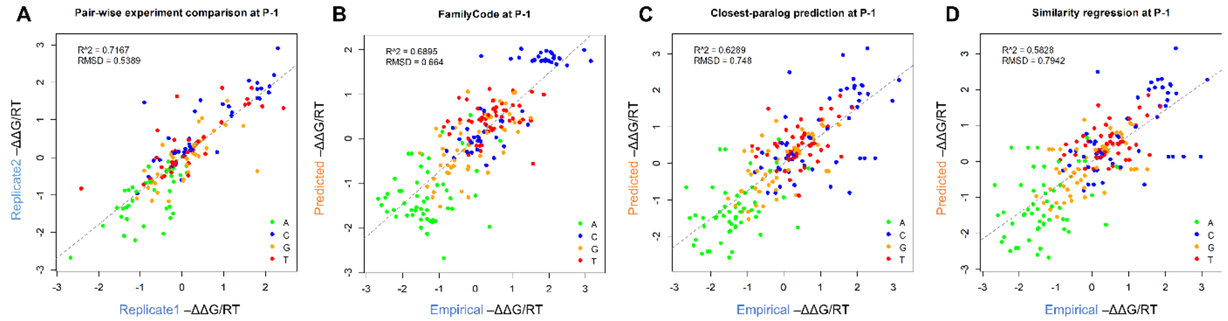

<u>Figure S5</u>: Cross-validation on wild-type bHLH binding motifs generated from HT-SELEX data. (**A**) Reproducibility between HT-SELEX replicates. (**B-D**) Comparison between empirically determined binding free energy parameters and predictions made using our family-code (**B**), the closest-paralog method (**C**), or similarity regression (**D**).

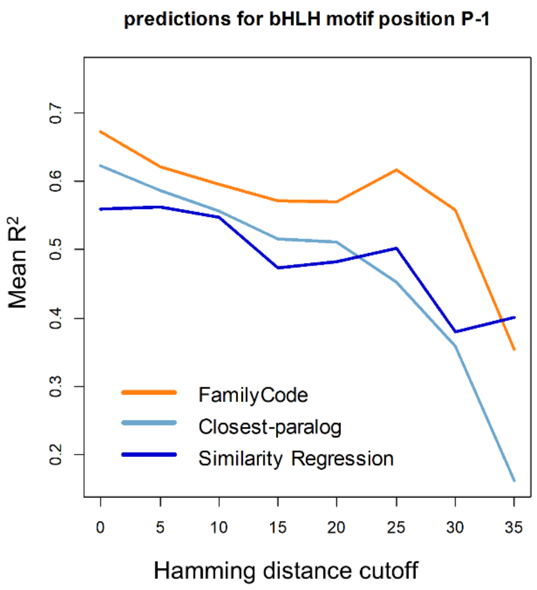

<u>Figure S6</u>: Performance of leave-one-out cross-validation at various hamming distance thresholds between held-out TF and training set TFs. Results are shown for bHLH models derived from HT-SELEX.

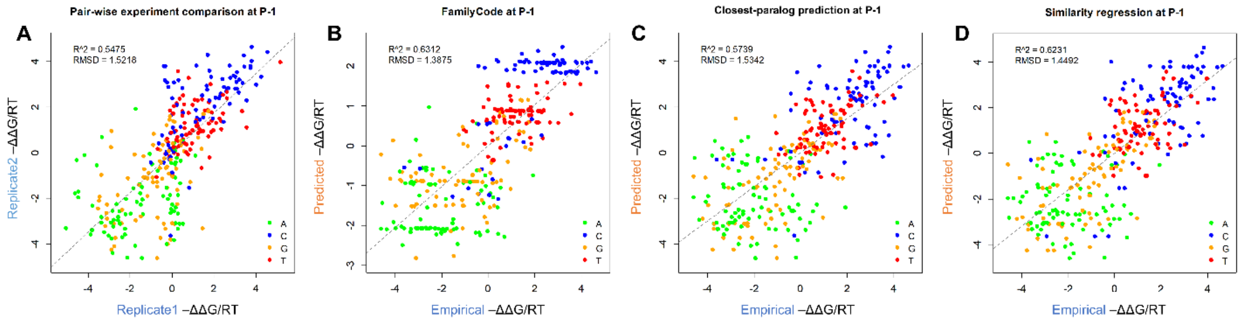

<u>Figure S7</u>: Cross-validation on wild-type bHLH binding motifs generated from PBM data. (**A**) Reproducibility between PBM replicates. (**B-D**) Comparison between empirically determined binding free energies and predictions made using our family-code (**B**), the closest-paralog method (**C**), or similarity regression (**D**).

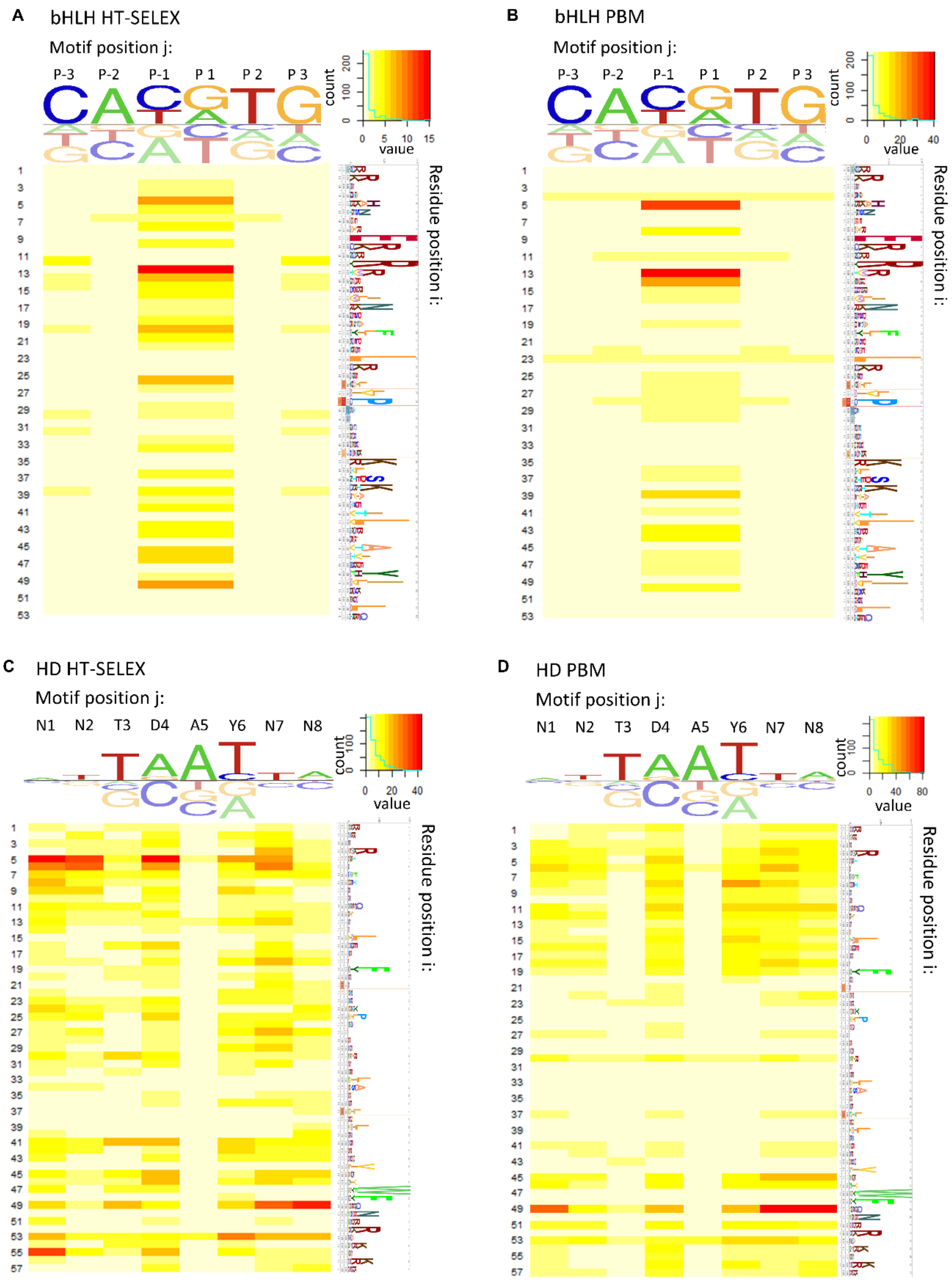

<u>Figure S8</u>: Positional association maps. Shown are results for the bHLH (**A**, **B**) and HD (**C**, **D**) families generated using HT-SELEX (**A**, **C**) and PBM (**B**, **D**) data.

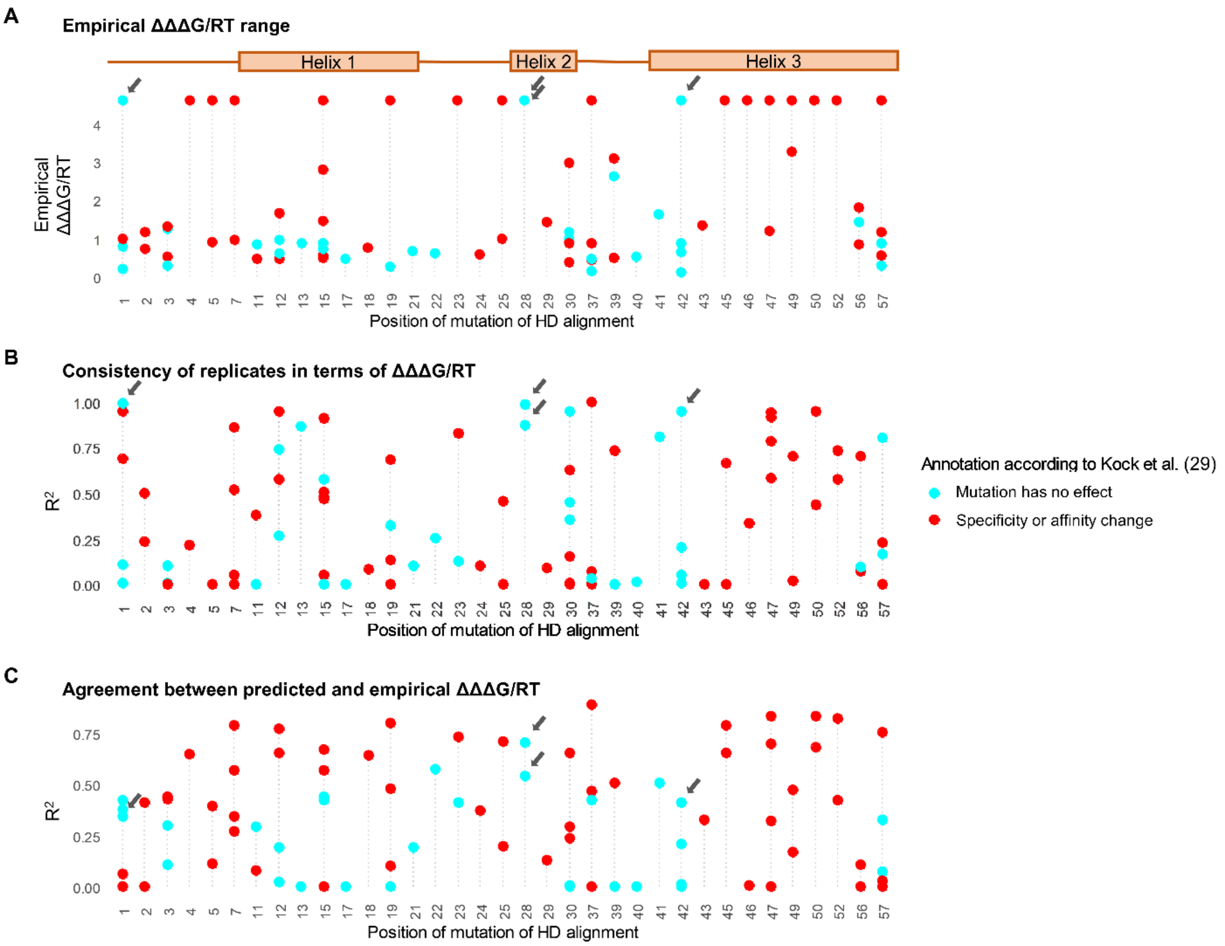

Figure S9: Reanalysis of PBM data for a set of 92 mutant homeodomains. (A) ΔΔΔG/RT range of empirical motif model plotted along mutation position. Wilcoxon signed-rank test of ΔΔΔG/RT ranges between reported change and unchanged sample resulted in p = 0.00063. (B) Empirical R^2 between two replicates of each mutant. (C) Prediction R^2 between FamilyCode prediction ΔΔΔG model and replicate 1 of empirical model. Gray arrows denote mutations that have specificity-determining effects that were not reported in the original study (29).

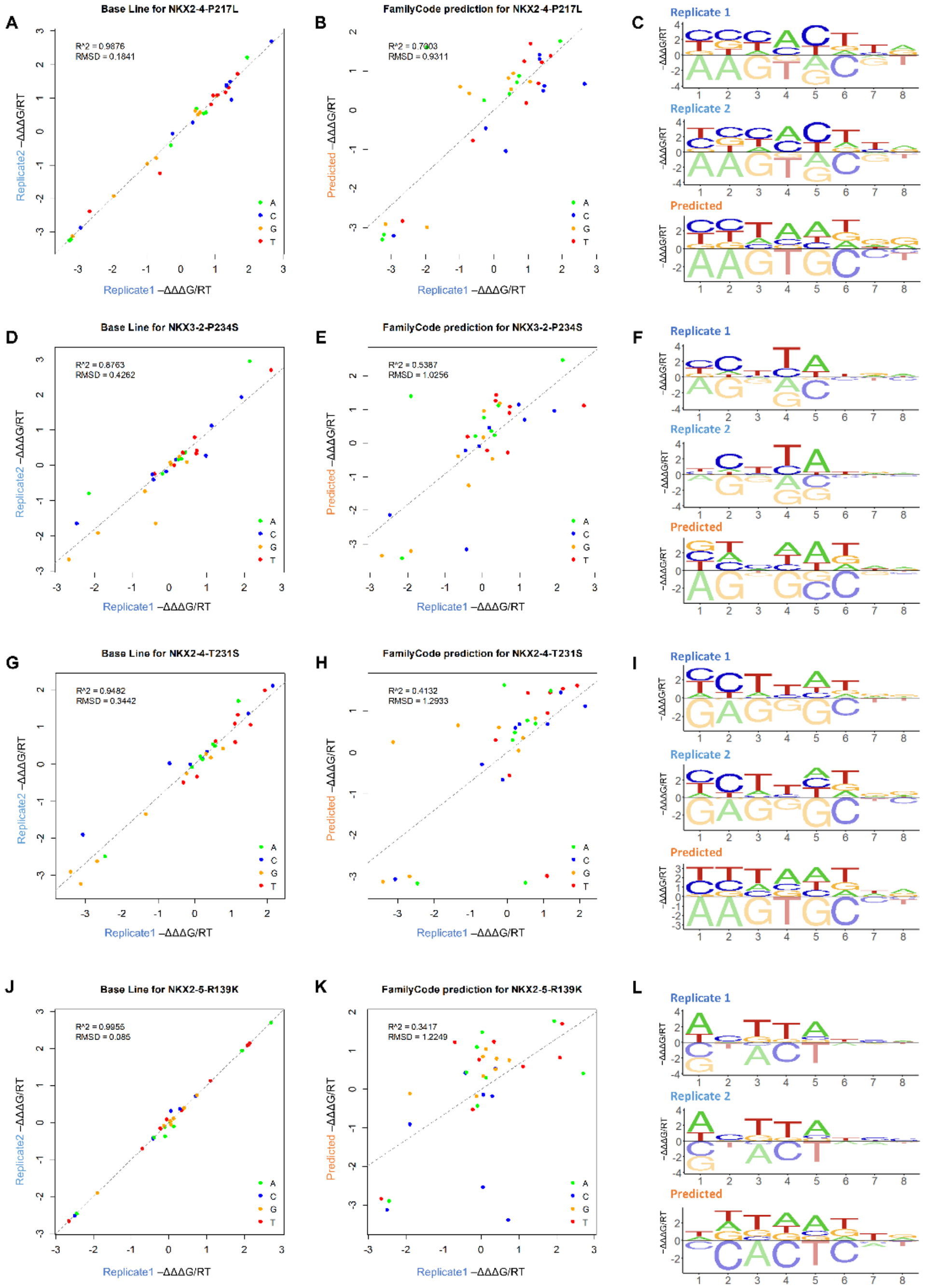

Figure S10: Examples of predictions of DNA binding specificity changes for mutant homeodomains. (**A**) FamilyCode prediction vs. replicate 1 for NKX2.4-P217L (P28L). (**B**) Replicate 1 vs. replicate 2 for NKX2.4-P217L (P28L). (**C**) Empirical and predicted ΔΔΔG models for NKX3.2-P234S (P28S). (**D**) FamilyCode prediction vs. replicate 1 for NKX3.2-P234S (P28S). (**E**) Replicate 1 vs. replicate 2 for NKX3.2-P234S (P28S). (**F**) Empirical and predicted ΔΔΔG models for NKX3.2-P234S (P28S). (**G**) FamilyCode prediction vs. replicate 1 for NKX2.4-T231S (T42S). (**H**) Replicate 1 vs. replicate 2 for NKX2.4-T231S (T42S). (**I**) Empirical and predicted ΔΔΔG models for NKX2.4-T231S (T42S). (**J**) FamilyCode prediction vs. replicate 1 for NKX2.5-R139K (R1K). (**K**) Replicate 1 vs. replicate 2 for NKX2.5-R139K (R1K). (**L**) Empirical and predicted ΔΔΔG models for NKX2.5-R139K (R1K).

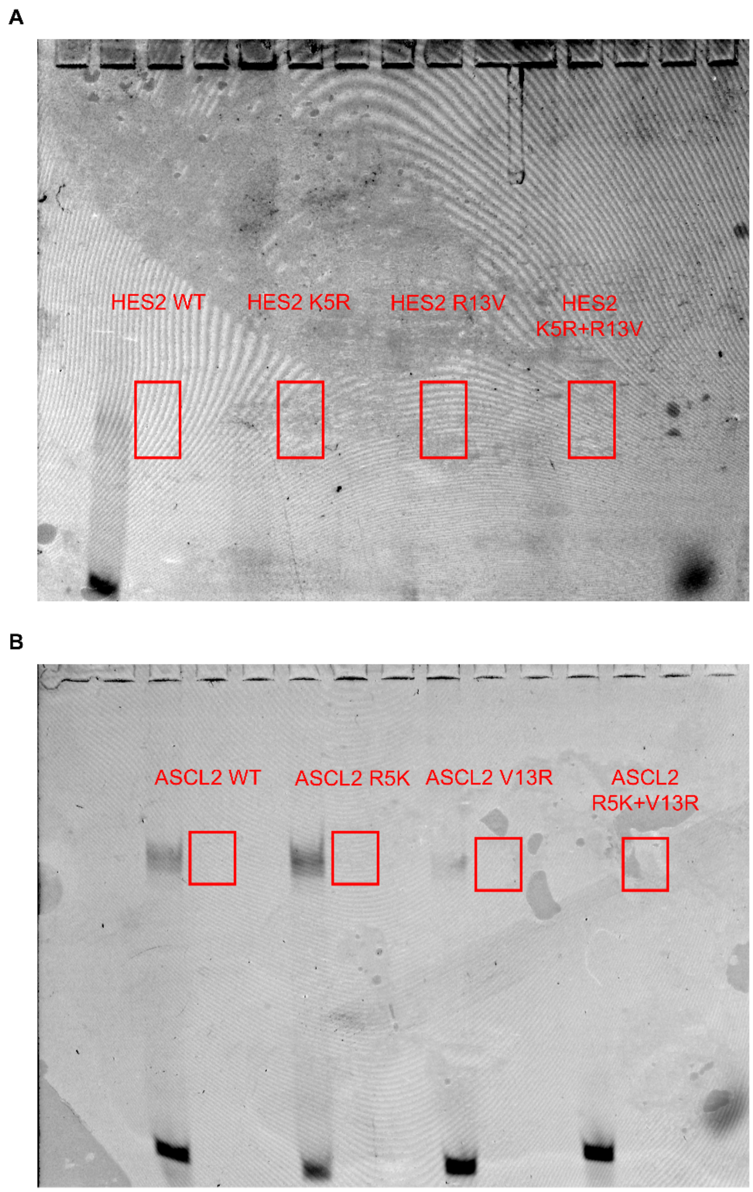

<u>Figure S11</u>: Raw EMSA gels. Shown are the bands we cut out to perform SELEX-seq for wild-type and mutant HES2 (**A**) and ASCL2 (**B**).
